# Convergent evolution of plant prickles by repeated gene co-option over deep time

**DOI:** 10.1126/science.ado1663

**Published:** 2024-08-02

**Authors:** James W. Satterlee, David Alonso, Pietro Gramazio, Katharine M. Jenike, Jia He, Andrea Arrones, Gloria Villanueva, Mariola Plazas, Srividya Ramakrishnan, Matthias Benoit, Iacopo Gentile, Anat Hendelman, Hagai Shohat, Blaine Fitzgerald, Gina M. Robitaille, Yumi Green, Kerry Swartwood, Michael J. Passalacqua, Edeline Gagnon, Rebecca Hilgenhof, Trevis D. Huggins, Georgia C. Eizenga, Amit Gur, Twan Rutten, Nils Stein, Shengrui Yao, Adrien Poncet, Clement Bellot, Amy Frary, Sandra Knapp, Mohammed Bendahmane, Tiina Särkinen, Jesse Gillis, Joyce Van Eck, Michael C. Schatz, Yuval Eshed, Jaime Prohens, Santiago Vilanova, Zachary B. Lippman

**Affiliations:** 1 Cold Spring Harbor Laboratory, Cold Spring Harbor, NY, USA.; 2 Howard Hughes Medical Institute, Cold Spring Harbor Laboratory, Cold Spring Harbor, NY, USA.; 3 Instituto de Conservación y Mejora de la Agrodiversidad Valenciana, Universitat Politècnica de València, Valencia, Spain.; 4 Department of Computer Science, Johns Hopkins University, Baltimore, MD, USA.; 5 Department of Genetic Medicine, Johns Hopkins University, Baltimore, MD, USA.; 6 French National Institute for Agriculture, Food, and Environment, Laboratory of Plant-Microbe Interactions, Toulouse, France.; 7 School of Biological Sciences, Cold Spring Harbor Laboratory, Cold Spring Harbor, NY, USA.; 8 Boyce Thompson Institute, Ithaca, New York, USA.; 9 Department of Integrative Biology, University of Guelph, Ontario, Canada.; 10 Royal Botanic Garden Edinburgh, Edinburgh, UK.; 11 USDA-ARS, Dale Bumpers National Rice Research Center, Stuttgart, AR, USA.; 12 Cucurbits Section, Department of Vegetable Sciences, Agricultural Research Organization, Newe Ya’ar Research Center, Ramat Yishay, Israel.; 13 Leibniz Institute of Plant Genetics and Crop Plant Research, Gatersleben, Germany.; 14 Crop Plant Genetics, Martin Luther University of Halle-Wittenberg, Halle (Saale), Germany.; 15 Department of Plant and Environmental Sciences, New Mexico State University, Las Cruces, NM, USA.; 16 Sustainable Agriculture Sciences Center, New Mexico State University, Alcalde, NM, USA.; 17 Laboratoire Reproduction et Developpement des Plantes, INRAE, CNRS, Universite Lyon, Ecole Normale Superieure de Lyon, Lyon, France.; 18 Department of Biological Sciences, Mount Holyoke College, South Hadley, MA, USA.; 19 Natural History Museum, London, UK.; 20 Physiology Department and Donnelly Centre for Cellular and Biomolecular Research, University of Toronto, Toronto, Ontario, Canada.; 21 Plant Breeding and Genetics Section, Cornell University, Ithaca, NY USA.; 22 Department of Plant and Environmental Sciences, The Weizmann Institute of Science, Rehovot, Israel.

## Abstract

An enduring question in evolutionary biology concerns the degree to which episodes of convergent trait evolution depend on the same genetic programs, particularly over long timescales. Here we genetically dissected repeated origins and losses of prickles, sharp epidermal projections, that convergently evolved in numerous plant lineages. Mutations in a cytokinin hormone biosynthetic gene caused at least 16 independent losses of prickles in eggplants and wild relatives in the genus *Solanum*. Homologs underlie prickle formation across angiosperms that collectively diverged over 150 million years ago, including rice and rose. By developing new *Solanum* genetic systems, we leveraged this discovery to eliminate prickles in a wild species and an indigenously foraged berry. Our findings implicate a shared hormone-activation genetic program underlying evolutionarily widespread and recurrent instances of plant morphological innovation.

## One-Sentence Summary

Uncovering a shared genetic basis for the convergent evolution of prickles facilitates their elimination in crop improvement.

Trait convergence, defined as the emergence of analogous traits in distantly related organisms, was a key observation made by Darwin in support of his theory of evolution. He recognized that similar selective pressures could lead to similar yet independently derived adaptations across species. However, the extent to which phenotypic convergence is driven by corresponding convergence in underlying genetic programs is poorly understood. Within a species, adaptive traits may arise from selection acting on standing genetic variation within and among populations, making phenotype-genotype convergence more likely (1, 2). At higher taxonomic levels and with increasing evolutionary divergence, phenotype-genotype convergence is posited to decline due to variation in allelic diversity, genomic background, and developmental mechanisms (3, 4). However, opportunities to dissect convergence at these timescales are scarce; finding convergent traits across wide evolutionary spans that are genetically tractable and well-supported by genomic data has remained a significant challenge.

**Fig. 1.**
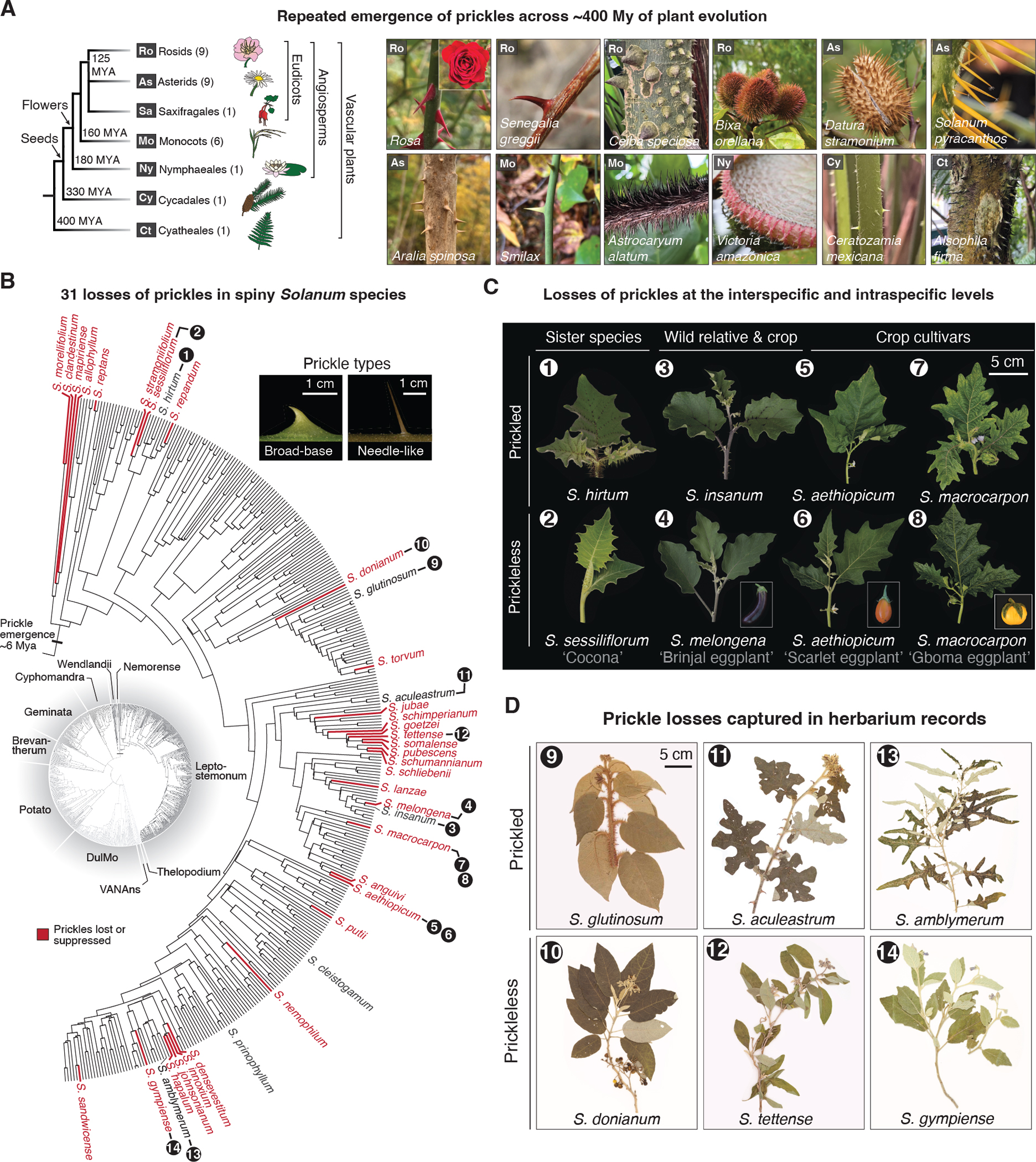
Prickles evolved convergently across vascular plants and were lost repeatedly in the spiny *Solanum* lineage. (**A**) Phylogeny, from (5), and corresponding images of representative vascular plants that independently evolved prickles. Number in parentheses indicates number of identified independent evolutionary origins of prickles (**B**) Phylogenetic tree [adapted from (10, 11)] of the spiny *Solanum* (subclades Wendlandii, Nemorense, and Leptostemonum) with species having lost prickles highlighted in red. Representative images of narrow and broad-based prickle morphologies are shown. (**C** and **D**) Images of *Solanum* taxa that have lost prickles captured from living (C) and herbarium (D) collections. Numbers correspond to species shown in (B).

In plants, sharp epidermal projections known as prickles convergently evolved at least 28 times over more than 400 million years of evolution (5) (Fig. 1A and Table S1). Prickles serve adaptive functions in herbivore deterrence, climbing growth, plant competition, and water retention (6–9). Rose (*Rosa* spp.) is a widely recognized taxon bearing prickles, though these prickles are vernacularly called thorns. True thorns, which are found on the trees of citrus (*Citrus* spp.) and honey locusts (*Gleditsia* spp.), for example, develop from axillary branches, whereas prickles originate from the epidermis or cortex, typically in association with hair-like structures known as trichomes (6). Despite their diverse adaptive roles and the broad phylogenetic diversity of their origins, prickles exhibit remarkable morphological similarity (Fig. S1A–C). Moreover, prickles have been lost or suppressed in numerous lineages. Therefore, prickle formation is an attractive system to determine whether episodes of repeated trait evolution rely on the same genetic programs over both short and long evolutionary timescales.

In the genus *Solanum*, which includes the major crops eggplant, potato, and tomato, prickles emerged in the common ancestor of the so-called “spiny *Solanums*” around 6 million years ago (Mya) (10, 11). This lineage includes the large Leptostemonum clade, which comprises hundreds of globally distributed species, including all cultivated eggplants and their wild progenitors (Fig. 1B). Prickle morphologies across the clade range from broad at the base (broad-based), or narrow-based and needle-like. Prickles occur on stems, along the vasculature of leaves, and on calyces, the outer whorl of floral organs. Several spiny *Solanum* species underwent human-driven selection for losses or suppression of prickles (12, 13), facilitating comparisons of prickled and prickleless sister species, crop species, and wild relatives (Fig. 1C and Table S2). An agriculturally significant instance of prickle loss occurred during the domestication of the widely cultivated Brinjal eggplant (*S. melongena*); however, prickle losses have also been observed in wild *Solanum* species without history of domestication (Fig. 1D). The specific genes controlling prickle development are unknown.

## Repeated losses of prickles in cultivated eggplants are caused by *LOG* gene mutations

**Fig 2.**
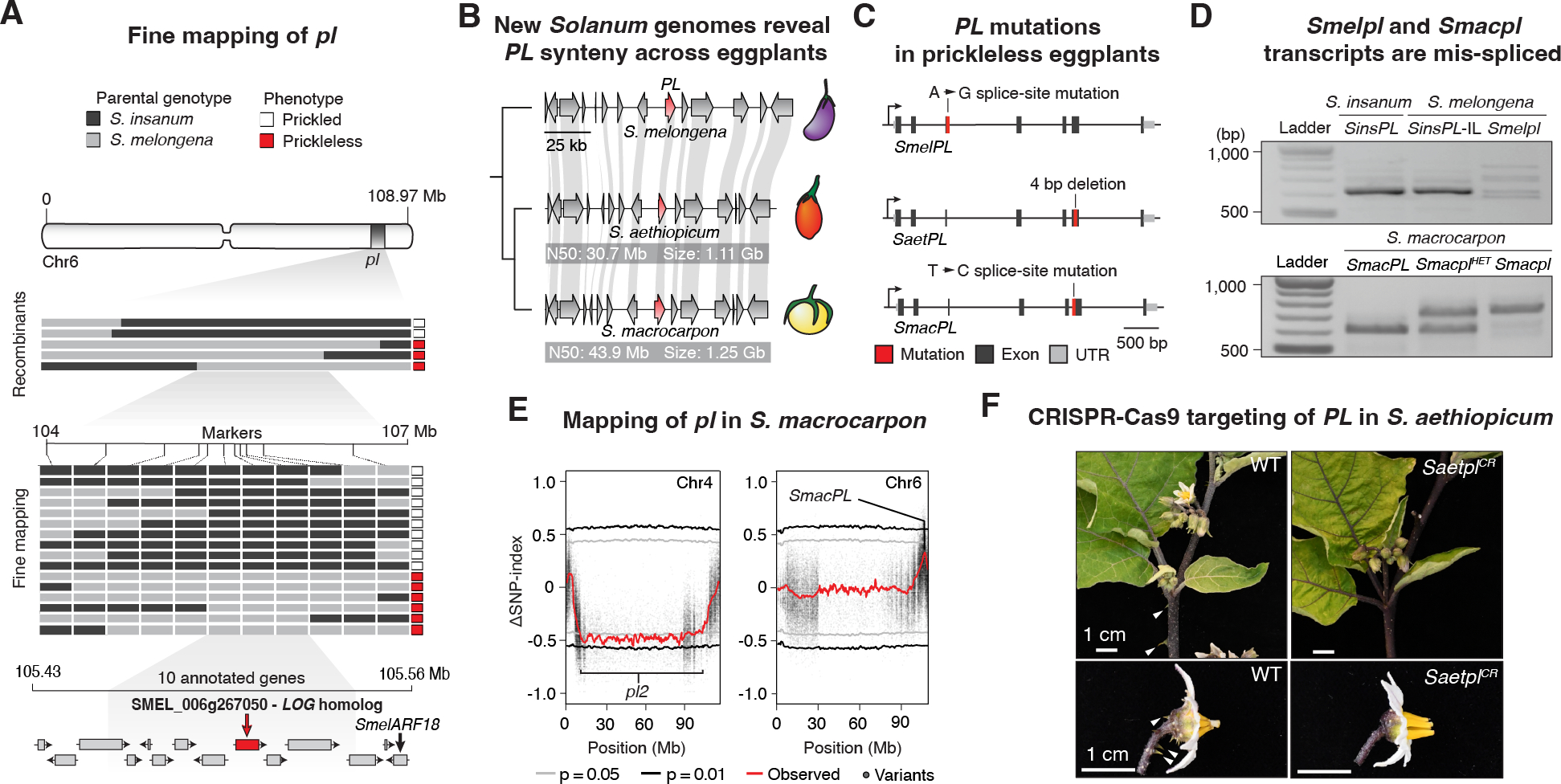
Losses of prickles in three domesticated *Solanum* species are caused by independent mutations in a *LOG* cytokinin biosynthetic gene. (**A**) Fine-mapping of *pl* in a Brinjal eggplant (*S. melongena*) x wild progenitor species (*S. insanum*) mapping population. (**B**) Genome sequencing and chromosome-scale assemblies of two African eggplants, the Scarlet eggplant (*S. aethiopicum*) and the Gboma eggplant (*S. macrocarpon*) reveals synteny of the *pl* locus. Genome summary statistics are indicated. (**C**) Independent mutations in a *LOG* gene in the *pl* interval in all three prickleless crop species. (**D**) Mis-splicing of *PL* transcripts caused by the *pl* mutations in Bringal eggplant *pl (Smelpl)* and Gboma eggplant *pl (Smacpl)* confirmed by RT-PCR. *SinsPL*-IL denotes an introgression of *S. insanum PL* into the Brinjal eggplant genomic background. (**E**) QTL-Seq identifies two loci that independently cause the prickleless phenotype in Gboma eggplant. (**F**) Phenotypes resulting from CRISPR-Cas9 genome editing of *SaetPL* in a prickled *S. aethiopicum* accession. Arrowheads indicate prickles.

Previous mapping studies in Brinjal eggplant showed that the loss of prickles is inherited as a single Mendelian locus designated *prickleless* (*pl*) and localized to a genomic interval on chromosome 6 (14). Using a recurrent backcross-derived mapping population between Brinjal eggplant and its prickled wild progenitor *S. insanum*, we confirmed this result and further fine-mapped *pl* to a ~100 kb interval containing 10 annotated genes (Fig. 2A). Just outside this interval is the previously proposed *pl* candidate gene *SmelARF18*, a putative auxin hormone response transcription factor (15). However, we did not find conspicuous coding region loss-of-function mutations in this gene or in any other gene in the interval. Instead, we identified a probable splice-site mutation in a gene encoding a LONELY GUY (LOG)-family cytokinin biosynthetic enzyme. LOG family members catalyze the final step in the biosynthesis of bioactive cytokinin, a hormone with roles in plant cell proliferation and differentiation (16). In a collection of 23 re-sequenced eggplant accessions (17), we found that this splice-site mutation was consistently associated with the prickleless phenotype, except in one accession, which harbored a 474 bp deletion in exon 6 of the *LOG* gene (Fig. S2A and Table S3).

The discovery of two independent mutations in the *LOG* candidate gene suggested that the loss of prickles occurred at least twice in Brinjal eggplant or its wild relatives. It also raised the possibility that mutations in orthologous genes may have caused parallel prickle losses in two other independently domesticated African eggplant species, the Scarlet eggplant (*S. aethiopicum*) and the Gboma eggplant (*S. macrocarpon*). Genomic resources for these indigenous crop species are limited. We therefore sequenced and assembled high-quality (QV ≥ 51, completeness > 99) chromosome-scale genomes and generated gene annotations for both species (Fig. 1C, Tables S4 and S5). Using these resources, we found that synteny within the *pl* locus was retained across all three cultivated eggplant species (Fig. 2B), and that prickleless Scarlet eggplant and Gboma eggplant each harbored different loss-of-function mutations in their respective *LOG* orthologs (Fig. 2C). Scarlet eggplant carries an indel mutation leading to a frameshift in the coding sequence and a prematurely terminated protein product, while Gboma eggplant carries a splice-site mutation. Reverse-transcriptase polymerase chain reaction (RT-PCR) on cDNA revealed lower expression and multiple mis-spliced transcripts in Brinjal eggplant and a mis-spliced isoform with a retained intron in Gboma eggplant (Fig. 2D). PCR sequencing revealed these transcripts were non-functional (Fig. S2B–D).

To further validate that these independent mutations explain the prickleless phenotypes, we next performed co-segregation analysis in F2 populations derived from intraspecific crosses between prickled and prickleless parents (Fig. 1C and S2). In Scarlet eggplant, homozygosity of the *LOG* mutant allele co-segregated with the prickleless phenotype in a Mendelian recessive fashion in all examined individuals (χ^2^ = 0.52, df = 1, *p* = 0.47). In Gboma eggplant we observed segregation patterns that indicated the presence of another unlinked recessive variant independently contributing to prickle loss (χ^2^ = 14.8, df = 1, *p* < 0.001). Leveraging our newly developed genomic resources, we used a mapping-by-sequencing approach to identify a second large interval associated with the loss of prickles on chromosome 4, which we designated *pl2* (Fig. 2E). Importantly, all segregating homozygous mutant individuals at *pl* on chromosome 6 carried the *LOG* gene splice-site mutation, although this genotype class was represented at lower-than-expected frequency, likely owing to segregation distortion (Fig. S2G). Finally, we modified existing plant regeneration and transformation protocols to engineer loss-of-function *PL* alleles using CRISPR-Cas9 genome editing in a prickled accession of *S. aethiopicum*. Analysis of three independently edited multiallelic transformants revealed suppression of prickle development due to numerous frameshift mutations resulting in *PL* loss-of-function (Fig. 2F and S2F). Transformants lacking mutations retained prickles. Taken together, these results indicate that *PL* is the *LOG* candidate gene, and that at least four independent mutations in this gene enabled repeated selection for losses of prickles in cultivated eggplant species.

## Mutations in *PL* are found in prickleless wild and cultivated species across the *Solanum*

The clade encompassing all three of the cultivated eggplants diverged ~2 Mya, but prickles in *Solanum* are more ancient, having emerged over ~6 Mya, and 31 independent losses of prickles have been documented, including in additional domesticated and wild species (11). We tested whether mutations in *PL* underlie these repeated instances of prickle loss across this broader evolutionary timescale by sampling DNA from additional prickleless species and their prickled close relatives. Because many wild *Solanum* species are too rare or geographically inaccessible for live-tissue sampling, we used a combination of PCR-amplified exon sequencing from herbarium tissue samples and whole-gene sequencing from available live tissue samples to detect *PL* mutations (Fig. S3 and Table S6).

**Fig. 3.**
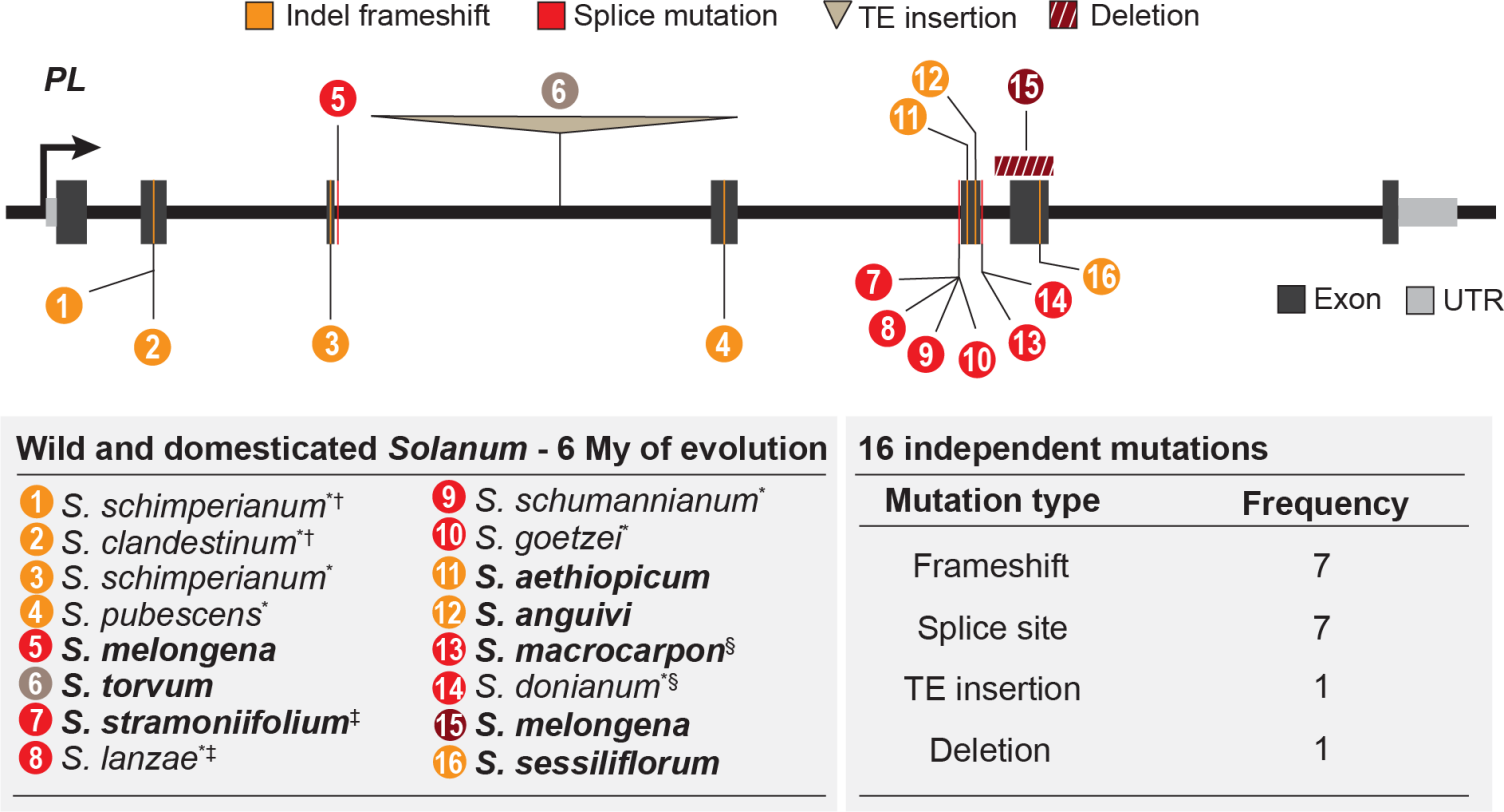
Mutations in *PL* are associated with prickle suppression across the spiny *Solanum*. *PL* variants with strong probable deleterious effects on gene function identified in prickle-suppressed taxa but not in closely-related prickled sister taxa. Mutations are numbered and shown along with their corresponding species name and sample source in the table below. In the tables, bold text indicates cultivated species, (*) indicates that genotyping was performed on archival herbarium samples, (†,‡,§) indicate species pairs that share identical but not necessarily ancestral mutations.

Along with the four *PL* mutations identified in our analysis of prickleless eggplants, we identified an additional 12 allelic mutations predicted to deleteriously affect *PL* function at the pan-genus level across the spiny *Solanum* (Fig. 3). These mutations, together with those detected by mapping, were associated with 14 out of the 31 recorded losses of prickles across the genus at the species level (Fig. 1B and Table S7). We then confirmed that these mutations were not found in prickled species from closely-related lineages (Fig. S3). In some cases, we detected the same, although not necessarily ancestrally derived, alleles in separate species. For example, the same splice-site mutation found in prickleless Gboma eggplant, native to and cultivated almost exclusively in Africa, was also identified in the wild species *S. donianum*, whose native range is in Central America and the Caribbean. Likewise, an identical splice-site mutation was found in both the wild species *S. lanzae*, from western Africa, and the foraged and sometimes cultivated species *S. stramoniifolium*, native to northern South America. Such genetic convergence at the allelic level may reflect the high penetrance of *PL* splicing defects, which can be conferred by mutationally accessible single nucleotide variants (18). Together, our results suggest that *PL* had an important and repeated genetic role in the convergent losses of prickles across *Solanum* in the wild and in cultivation. However, loci other than *PL* may explain prickles losses in lineages for which mutations were not identified.

## Repeated co-option of *LOG* homologs underlies prickle convergent evolution

**Fig. 4.**
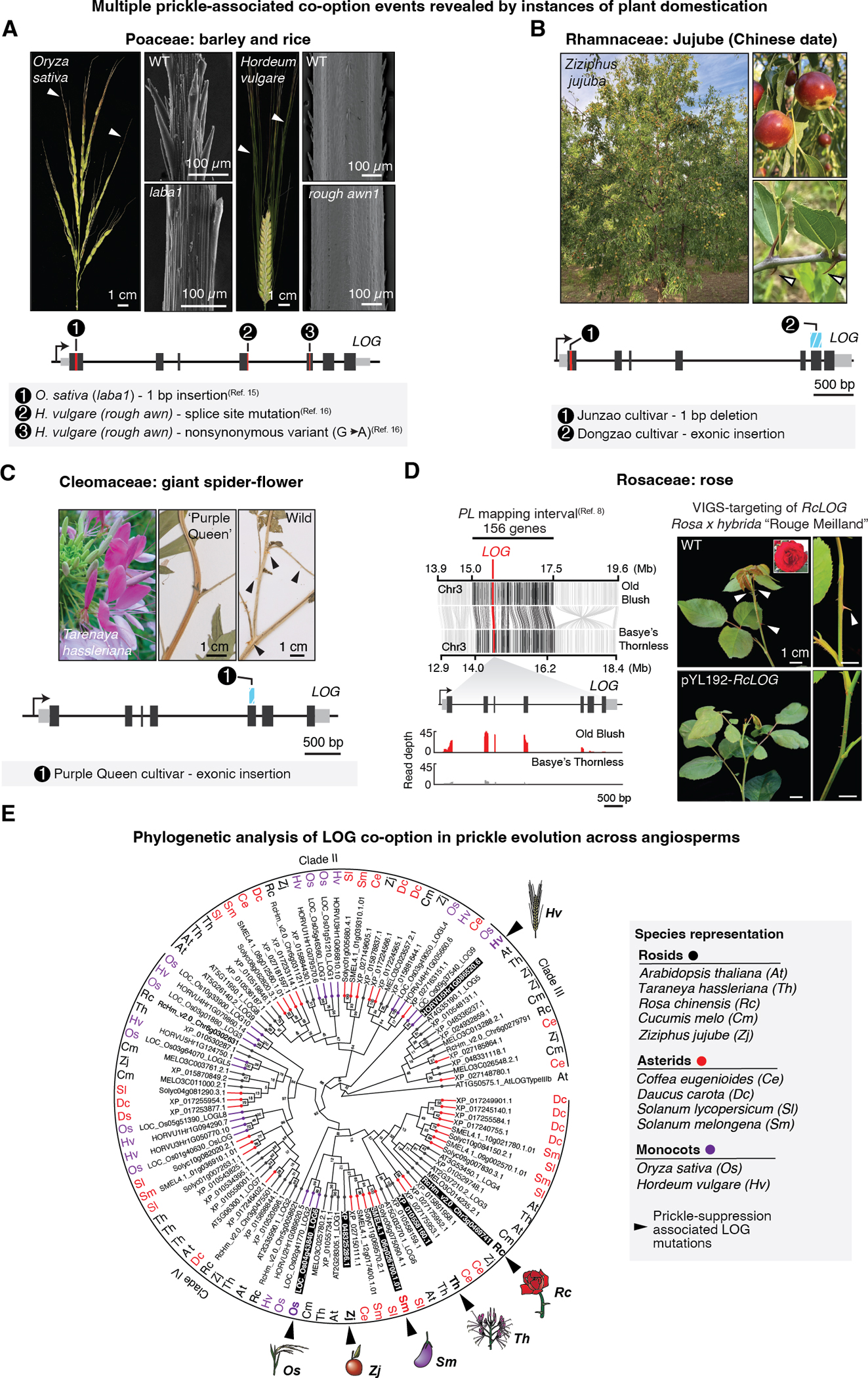
Losses of convergently evolved prickles across angiosperms are associated with *LOG* mutations. (**A** to **D**) Instances of prickle suppression in angiosperms associated with *LOG* mutations depicted in corresponding *LOG* gene diagrams. (A) Images of rice and barley WT inflorescences. Arrowheads indicate awns, which are shown for WT and mutant genotypes (rice, *laba1*; barley, *rough awn1*) by SEM. (B) Images of jujube trees, fruits, and stipular spines (arrowheads). Two less spiny cultivated varieties harbor two independent *LOG* mutations. (C) The ornamental giant spider flower (pictured) carries a mutated *LOG* gene in the sequenced ‘Purple Queen’ cultivar. Cultivated varieties bear fewer smaller prickles (arrowheads) than wild varieties, as reflected in herbarium samples. (D) (Left) Loss of prickles in rose maps to a ~2.5 Mb interval harboring a *LOG* gene with severely reduced expression in the prickleless cultivar relative to the prickled cultivar. Syntenic genes within the mapping interval of the prickled ‘Old Blush’ and prickleless ‘Basye’s Thornless’ parental lines are shown in black. Read pileups show average *LOG* expression in leaves of the parental genotypes (*N* = 3). (Right) VIGS targeting of the candidate *LOG* gene leads to suppression of prickles in an ornamental rose hybrid. (**E**) Protein-based phylogenetic tree of the Arabidopsis LOG1 orthogroup defined by Orthofinder, from the indicated asterid (red), rosid (black), and monocot (purple) species. LOGs encoded by genes with mutations in prickle-suppressed taxa are indicated by arrowheads.

The finding of recurrent mutations in *PL* orthologs across the spiny *Solanums* suggested that co-option of cytokinin biosynthetic gene function was critical to prickle evolution. This spurred us to ask whether genetic convergence through *LOG* gene co-option extends to other prickled species across flowering plants. We searched the literature for studies associating instances of loss or suppression of sharp outgrowths with specific genomic loci or genes. Strikingly, we found that in the grass family (Poaceae) independent mutant alleles in a *LOG* homolog from rice (*Oryza sativa*) and barley (*Hordeum vulgare*) conferred near complete suppression of epidermally-derived sharp projections commonly called “barbs” but botanically classified as prickles (19, 20). In contrast to the conspicuous, multi-cellular, and lignified prickles found in the *Solanum* (Fig. S1), grass prickles are homologous structures made of silicified single-cells that develop on awns (Fig. 4A), an outer-whorl structure of the grass flower involved in seed dispersal, along with leaves and spikelets.

Mining additional genomic data for *LOG* mutations co-occurring with losses of prickles in other eudicot lineages, we found that the fruit-bearing tree crop jujube, commonly known as Chinese date (*Ziziphus jujuba*), in the Rhamnaceae family, carried two independent mutations (a 1 bp deletion and an exonic insertion) in a *LOG* homolog in two cultivars with suppressed prickles (also known as stipular spines) (Fig. 4B) (21–23). Importantly, neither mutation was found in Sour jujube (*Z. jujuba* var. *spinosa*), the prickled wild progenitor. We also detected an exonic insertion in a *LOG* homolog of the prickle suppressed ‘Purple Queen’ cultivar of giant spider flower (*Teranaya hassleriana*), a widely cultivated ornamental plant in the Cleomaceae, a small family within the Brassicales closely related to Arabidopsis (Fig. 4C and Table S6) (24). Finally, in rose, which is a commercially important cultivated cut flower, previous mapping for loci conferring “thornlessness” identified two major effect loci (9), as we found in *S. macrocarpon*. One of these was a ~2.5 Mb interval containing 156 annotated genes (Fig. 4D), which we found includes a *LOG* homolog. Though there were no obvious coding or splicing mutations in this *LOG*, we found that its expression was substantially reduced in the leaves of the mapping parent cultivar ‘Bayse’s thornless’ (*Rosa wichuraiana*) compared to the prickled parent *Rosa chinensis* (Fig. 4D). To determine whether this candidate *LOG* has a role in rose prickle development we used a virus induced gene silencing (VIGS) approach to reduce *LOG* function (25). In 2/14 rose plants infected with the tobacco rattle virus (TRV) expressing an inverted repeat of *LOG* RNA, strong suppression of prickle development was observed, while wild-type plants of the same background showed normal prickle development (Fig. 4D).

Taken together, these findings suggested that *LOG* gene reuse was critical in the independent acquisition of prickles in numerous plant lineages that last shared a common ancestor ~150 Mya. Most sequenced seed plants (angiosperms and gymnosperms) retain multiple *LOG* gene copies within their genomes. In these taxa, the mean number of annotated *LOG* genes is 15, inflated by recent polyploid lineages, while the median and mode copy numbers are 12 and 10, respectively (*N* = 160). To understand the phylogenetic context of *LOG* co-option and to ask whether repeated co-option occurs in a specific clade of *LOG* gene family members, we conducted an analysis of LOG family proteins from prickled and prickleless species across the angiosperms (Fig. 4E). Most of the prickle co-option associated LOGs occurred within a specific subclade of the LOG family, suggesting that co-option was more favorable in certain LOG family subclades, particularly those with lineage-specific duplications. However, the LOG homolog co-opted in barley is derived from an earlier diverging subclade (20), indicating that despite a subclade bias, co-option of other LOG family members in different clades may also be associated with prickle evolution.

## *LOG* gene diversification preceded *PL* co-option in *Solanum*

**Fig. 5.**
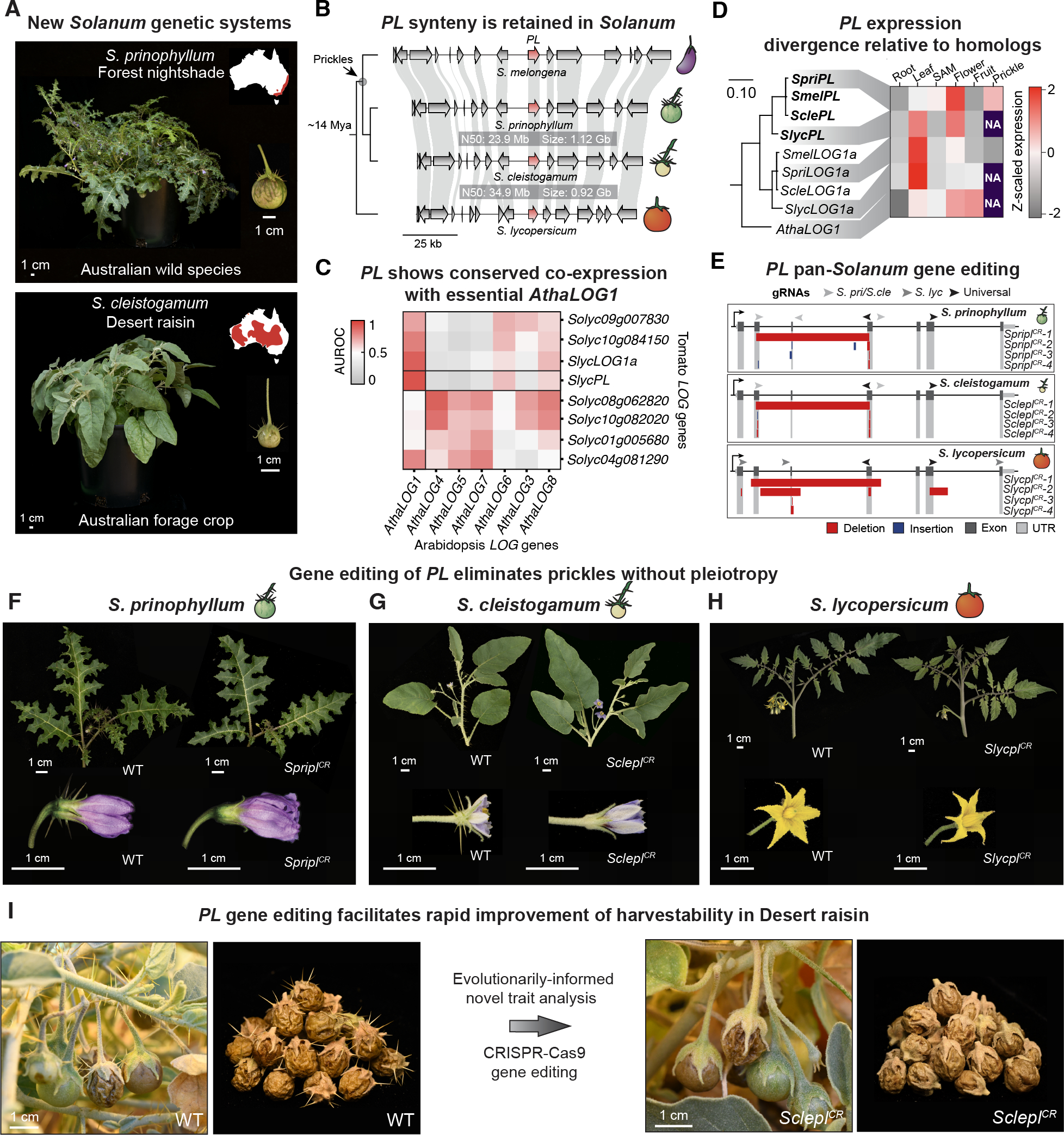
The *Solanum PL* gene was co-opted from an ancestral gene duplication event enabling non-pleiotropic editing of *PL* for crop improvement. (**A**) Whole-plant and fruit images of the prickled wild species Forest nightshade (*S. prinophyllum*, top) and its close foraged berry-producing relative Desert raisin (*S. cleistogamum*, bottom). Red-shaded region in map insets indicates approximate species ranges in Australia based on reported observations (http://www.flora.sa.gov.au/). (**B**) Genome sequencing and chromosome-scale assemblies of Forest nightshade and Desert raisin reveals that *PL* interval synteny is conserved in Brinjal eggplant and tomato (*S. lycopersicum*). Genome summary statistics are indicated. (**C**) Heatmap depicting the predictability of identifying cross-species co-expressed genes among cross-species pairs of *LOG* homologs based on their respective co-expression relationships in tomato and Arabidopsis. A higher Area Under the Receiver Operating Characteristic (AUROC) curve score indicates *LOG* homologs with increased conservation of their corresponding orthologous co-expressed genes. (**D**) Coding-sequence based maximum-likelihood phylogenetic tree of *Solanum PL* orthologs, their closely related paralog *LOG1a*, and *AthaLOG1* in comparable tissue types. Heatmap shows expression in matched tissues. (**E**) CRISPR-Cas9 gene editing strategy and resulting mutant alleles generated in Forest nightshade, Desert raisin, and tomato. (**F** to **H**) Phenotypes of WT and gene edited *pl* null mutants in Forest nightshade (F), Desert raisin (G), and tomato (H). Prickles are nearly completely suppressed (Forest nightshade) and eliminated (Desert raisin) obvious pleiotropic consequences. In tomato where *PL* was not co-opted for prickle development, *Slycpl*^*CR*^ mutants resemble wild type. (**I**) Evolutionarily-informed trait analysis enables rapid and expedient removal of prickles for improved harvestability in *Solanum* crops.

Given the recurrent co-option of *LOG* genes against a backdrop of paralogous gene family members, we sought to better understand the phylogenetic and genomic context that facilitated *LOG* co-option in *Solanum*. We examined the conservation of the *PL* locus, comparing the region across *Solanum*, including Brinjal eggplant and two additional spiny *Solanum* species, with tomato (*S. lycopersicum*), an ancestrally prickleless species that diverged prior to the evolution of the spiny *Solanums*. We first constructed high-quality chromosome-scale genome assemblies for *S. prinophyllum* (Forest nightshade; QV = 51.6, completeness > 99) and *S. cleistogamum* [Desert raisin; QV = 49.8, completeness > 99 (Tables S4 and S5)]. Forest nightshade is endemic to southeastern Australia whereas Desert raisin is native to the arid center of Australia and has been foraged by First Nations people for thousands of years for their sweet, dried berries (26) (Fig. 5A). In our screen for *PL* mutations across the spiny *Solanum* we did not identify any naturally occurring *PL* mutations in the Australian *Solanum* lineages to which these species belong (Fig. S3). Indeed, neither species has been domesticated and are distinct lineages from the cultivated eggplants.

Leveraging these newly developed genomic resources, we found that synteny at the *PL* locus was conserved across the *Solanum*, suggesting that *PL* was co-opted from a standing syntenic ortholog that existed at least since the divergence of tomato and the spiny *Solanums* ~14 Mya (Fig. 5B). To better understand ancestral *PL* function across eudicots, we performed a meta-analysis of gene expression data from Arabidopsis (3154 samples) and tomato (5491 samples), reasoning that shared expression profiles reflect the degree of shared inter-species function (27). We assessed each member of the *LOG* family for its ability to predict co-expression in every other member of the *LOG* family in the other species. An area under the receiver operating characteristic (AUROC) curve statistic of 0.93 indicated that *SlycPL* in tomato is co-expressed with nearly identical genes to that of *AthaLOG1* in Arabidopsis, pointing to a conserved function. Likewise, three other tomato *LOG* gene family members also exhibited strongly conserved co-expression with *AthaLOG1* (Fig. 5C). The *LOG1* clade, to which *PL* belongs, has therefore maintained signatures of functional conservation across ~120 My.

Tissue-specific knockdown of *AthaLOG1* in the Arabidopsis floral meristem has been shown to impair floral organ initiation, suggesting that *AthaLOG1* has critical roles in meristem maintenance, similar to the canonical developmental role for *LOGs* first reported in rice (16, 28). Therefore, duplication and diversification of the *LOG1* subclade in the *Solanum* may have facilitated *PL* functional co-option. To explore this hypothesis, we generated an expression atlas for prickled forest nightshade and compared it to matched-tissue gene expression data from tomato and Arabidopsis (Fig. 5D). In Arabidopsis, *AthaLOG1* possesses a broad expression pattern across tissues, while *Solanum PL* and *LOG1a* have evolved more tissue-biased expression patterns. Compared to its ortholog in tomato, forest nightshade *SpriPL* has evolved enriched expression in flowers and, consistent with its co-opted function, in developing prickles. Therefore, paralog diversification in the *Solanum* likely enabled functional co-option and redeployment of ancestral *LOG1* clade function in prickle development.

## Non-pleiotropic removal of prickles with gene editing

We reasoned that the co-option of *PL* could facilitate the engineering of agriculturally desirable loss-of-function prickleless mutants, even in the Australian spiny *Solanum* taxa in which we did not detect naturally occurring *PL* mutations. The duplication leading to *PL* and its subsequent expression divergence from its ancestral copy would prevent undesirable pleiotropic effects on other traits. Alternatively, cryptic background modifiers in prickleless lineages may have been required to specifically suppress prickle development, and thus eliminating *PL* would leave prickles intact, or result in pleiotropy. To distinguish between these two possibilities, we devised a pan-genus CRISPR-Cas9 editing strategy to target *PL* in Forest nightshade, Desert raisin, and tomato, the latter of which harbors a *PL* ortholog, likely performing an ancestral function outside of prickle development. Adapting techniques previously established in tomato (29), we developed plant regeneration, transformation, and genome editing for Forest nightshade and Desert raisin, thereby elevating these two species into new *Solanum* genetic systems. We engineered multiple loss-of-function mutations in *PL* (*pl*^*CR*^) in all three species and compared their phenotypes (Fig. 5E). In both Forest nightshade and Desert raisin, *pl*^*CR*^ individuals showed strong suppression of prickle development in all tissues and organs where prickles normally develop in wild type plants, though we observed small sporadic prickles (Figs. 5F,G and S4). Meanwhile, in tomato, *Slycpl*^*CR*^ plants resembled the wild type, likely due to genetic redundancy with *SlycLOG1a* and possibly other *LOG* family members prior to the *PL* co-option event ~6 Mya (Fig. 5H). Fruit morphology and sweetness remained unchanged (Brix sugar content ~30% compared to ~50% in grape raisins and ~9% in cherry tomato) and trichome density and morphology appeared unaffected in WT and *pl*^*CR*^ Desert raisin lines. These results suggest that *PL* targeting is an effective strategy for first line improvement of harvestability in wild or partially domesticated prickled species bearing edible fruits, including additional locally-important cultivated indigenous *Solanum* such as vila-vila (*S. sisymbriifolium*) and naranjilla (*S. quitoense*) (Fig. 5I, S5, and Table S8).

## Discussion

Here we showed multiple, phylogenetically independent reuses of *LOG* family members in prickle development across 150 My of plant evolution. Studies addressing convergent trait evolution at these timescales have hinted that similar and divergent genetic programs can underpin phenotypic convergence (3, 30). For example, the convergent evolution of echolocation in bats and cetaceans is associated with positive selection on variation in shared orthologous genes (31). In plants, convergent evolution of floral asymmetry has been shown in numerous species to occur by modified expression of the transcription factor encoding gene *CYCLOIDEA* (32). On the other hand, different loci were reported to underlie convergent adaptation to marine habitats in mammals (33). The repeated use of the same genetic program seen in some traits such as prickles may in part be due to their relative simplicity. Unlike composite traits (34), where selection has the potential to act on many different loci affecting many different organismal systems, convergent traits that arise from selection on fewer potentially relevant loci may exhibit greater genetic convergence by virtue of sheer probability. However, even traits of modest complexity, such as animal eye lenses composed of homomeric crystallins (35), can have many distinct genetic origins, indicating that trait complexity alone cannot fully account for observed patterns of convergent evolution.

Genotype-phenotype convergence may also rely on developmental constraints imposed on morphological innovation, which often depends on the re-purposing of ancestral genetic mechanisms (36, 37). Gene co-option may allow key developmental regulators to take on new roles via non-pleiotropically partitioning gene function, particularly when standing paralog diversity exists. This has been suggested as an explanation for the repeated evolution of limbs, for example, by co-option of *Hox* genes. We suggest that functionally redundant *LOG* paralogs that arose through lineage-specific or shared ancestral duplication events may acquire specialized functions, as we found with prickles. The lack of an apparent *pl* mutant phenotype in tomato, coupled with the strong suppression of prickles in *pl* mutants in prickled lineages without obvious effects on other traits in consistent with *PL* functional co-option. Even after co-option in prickle development, *LOG*s may retain some functional redundancy, as engineered and natural (i.e. rice and barley) *LOG* mutants still produce sporadic small prickles (Figs. 4A and S4). Even partial paralog redundancy may increase the odds of phenotype-genotype convergence by allowing selection for gains and losses of prickles while avoiding developmental pleiotropy.

Perhaps most importantly, as an essential plant hormone with key developmental functions, cytokinin is well-suited to serve a recurrent role in morphological adaptation. Like the plant hormones auxin and florigen, cytokinins have cell-type and stage-specific effects. For example, beyond its role in promoting cell proliferation in shoot meristems (16), cytokinin contributes to microtubule reorientation in maturing root epidermal cells (38) and promotes growth cessation associated with cell wall stiffening in the root differentiation zone (39). The results presented here endow cytokinin activation by LOGs with a central and repeated role in morphological innovation. This could occur by canonical cytokinin activation of cell proliferation but could also involve cytokinin promotion of the differentiation program leading to the hard, lignified structure of the prickle. Other plant morphological innovations are also controlled by cytokinin-related gene activity. Overexpression of a *LOG* gene is sufficient to induce the ectopic formation of shoot-borne tubers in axillary meristems in tomato (40), while a dominant mutation in a gene encoding a cytokinin receptor protein induces the ectopic formation of root nodules in the legume *Lotus japonicus* (41), both of which depend on localized cell proliferation. Unlike typical “master” regulators that often coordinate complex programs, such as floral homeotic genes (42), the repeated loss of prickles reported here relies on an enzymatic gene family involved in the activation of several types of cytokinins. Whether redeployment of such hormone activation genes in new developmental contexts is sufficient to generate morphological novelty warrants further study.

Finally, we propose that targeted gene editing of cytokinin biosynthesis and signaling components, as demonstrated here, is likely a predictable and efficient strategy for eliminating prickles in various flowering plant lineages. This approach is particularly promising for roses, where the labor intensive, manual removal of prickles is a common practice for most cut varieties. Though roses have variable ploidy (43) and genome editing in elite germplasm can be challenging (44), we demonstrated this potential using VIGS to suppress prickle development (Fig. 4D). Beyond the species presented here, the observed subclade bias in *LOG* homolog co-option will likely aid in selection of *LOG* genes for site-directed mutagenesis in other taxa. However, in principle, the general role of LOG proteins in cytokinin activation could allow more distantly related *LOG*s to carry out their role in prickle development, as occurs in barley (Fig. 4A,E). This necessitates consideration of both *LOG* gene expression and phylogenetic context for targeting prioritization. Overall, continued efforts to unite genetics, genomics, and genome editing across diverse plants, as illustrated in this study, will both advance our ability to track evolutionary changes over a broad range of time scales and empower the engineering of novel phenotypes to expand our use of plant diversity in agriculture.

## Methods Summary

For the mapping of *pl* in *S. melongena*, previously generated introgressions of prickled *S. insanum* into the prickleless *S. melongena* background were screened for small prickle-associated genomic intervals on chromosome 6 (45). An individual with the narrowest identified interval was then selfed and a total of 622 resulting progeny were used for fine mapping of *pl* by PCR-based marker genotyping.

For genome assembly, high molecular weight DNA was extracted from flash-frozen, dark-treated 4-week-old seedlings. A combination of long-read sequencing (Pacific Biosciences, CA, USA) and optical mapping (Bionano Genomics, CA, USA) data were used for assembly. Sequencing reads from each sample were assembled with hifiasm (46) exact parameters and software version varied between samples based on the level of estimated heterozygosity and are reported in Table S4. In addition, high-throughput chromosome conformation capture (Arima Genomics, CA, USA) was performed for one sample, *S. prinophyllum,* to finalize scaffolding. Using merqury (47), the final consensus quality (QV) of the assemblies was 51.1, on average.

For genome annotation, orthologs with coverage above 50% and 75% identity were lifted from Heinz v4.0 Heinz v4.0 (48) and Eggplant v4.1 (17) via Liftoff (49) and refined using protein and gene microsynteny support. The completeness of the gene models was determined by assessing single-copy orthologs using BUSCO5 (50).

Plant regeneration and *Agrobacterium tumefaciens*-mediated transformation of *S*. *prinophyllum* and tomato were performed according to (51). The same methods were also used for *S. aethiopicum* and *S. cleistogamum* with two modifications. For *S. cleistogamum*, plant regeneration, the medium was supplemented with 0.5 mg/L zeatin instead of 2 mg/L and for the selection medium, 75 mg/L kanamycin was used instead of 200 mg/L. For *S. aethiopicum*, the protocol was the same as for *S. cleistogamum*, except the fourth transfer of transformed plantlets was performed onto media supplemented with 50 mg/L kanamycin.

## Supplementary Material

Supplementary_tables

Supplementary Materials

Figure_S3

Figure_S1

Figure_S4

Figure_S2

Figure_S5

## Data Availability

Short-read sequencing data and genome assemblies reported in this paper are deposited at the NCBI Short Read Archive under BioProject ID PRJNA1073673. All other data are available in the main text or the supplementary materials. Plant materials used in this study are available upon request.
