# Supplementary material for: Convergent evolution of plant prickles by repeated gene co-option over deep time
Source: Science. Author manuscript; Available in PMC 2024 Aug 7. (PMC11305333; doi:10.1126/science.ado1663)
Supplement: Supplementary Materials [file NIHMS2012043-supplement-Supplementary_Materials.docx]

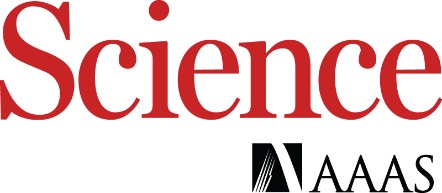


Supplementary Materials for

**Convergent evolution of plant prickles by repeated gene co-option over deep time**

**Authors:** James W. Satterlee^1,2^, David Alonso^3^, Pietro Gramazio^3^, Katharine M. Jenike^4,5^, Jia He^1,2^, Andrea Arrones^3^, Gloria Villanueva^3^, Mariola Plazas^3^, Srividya Ramakrishnan^4,5^, Matthias Benoit^6^, Iacopo Gentile^7^, Anat Hendelman^1,2^, Hagai Shohat^1^, Blaine Fitzgerald^1,2^, Gina M. Robitaille^1,2^, Yumi Green^8^, Kerry Swartwood^8^, Michael J. Passalacqua^7^, Edeline Gagnon^9,10^, Rebecca Hilgenhof^10^, Trevis D. Huggins^11^, Georgia C. Eizenga^11^, Amit Gur^12^, Twan Rutten^13^, Nils Stein^13,14^, Shengrui Yao^15,16^, Adrien Poncet^17^, Clement Bellot^17^, Amy Frary^18^, Sandra Knapp^19^, Mohammed Bendahmane^17^, Tiina Särkinen^10^, Jesse Gillis^1,20^, Joyce Van Eck^8,21^, Michael C. Schatz^4,5^, Yuval Eshed^22^, Jaime Prohens^3^, Santiago Vilanova^3^, Zachary B. Lippman^1,2,7*^

Corresponding author: [lippman@cshl.edu](mailto:lippman@cshl.edu)

**The PDF file includes:**

Materials and Methods

Figs. S1 to S5
Tables S1 to S9

References *52*-*97*

Materials and Methods

Plant growth conditions

Brinjal eggplant and wild eggplant (*Solanum insanum*) used for the mapping of *pl* were grown in a growth chamber under 25 °C/18 °C day/night temperatures and a 16/8h light/dark photoperiod, following the protocol for wild materials described in (*52*). The other eggplant species used for mapping and phenotyping were sown and germinated in 96-cell flats in a greenhouse, under natural and supplemental ~250 µmol m^-2^ s^-1^ light from high-pressure sodium bulbs (16 h light, 8 h dark) at daytime temperatures between 26-28 °C and night-time temperatures between 18-20 °C with a relative humidity of 40-60%. At ~4 weeks post-germination, seedlings were transplanted to 4 L pots containing high drainage PRO-MIX HP Mycorrhizae Growing Mix soil (Pro-Mix, PA, USA) supplemented with Osmocote fertilizer. Prickle phenotypes recorded for co-segregation analysis were performed in the field for the Scarlet eggplant (*S. aethiopicum*) and both the field (West Neck Field, Lloyd Harbor, NY, USA) and greenhouse for Gboma eggplant (*S. macrocarpon*). Field conditions included drip irrigation, regular soil fertilization, and manual weeding. All other *Solanum* species used in this study were grown in either the field or greenhouse, as specified (see **Table S6** for accession information).

Other plants imaged for this study were grown as follows. Rose plants were grown in the field or grown in an environmentally controlled greenhouse conditions at the Ecole Normale Supérieure of Lyon, France with 16 h light, 8 h dark day/night periods and 25 °C/19 °C day/night temperatures. Chinese date (*Z. jujuba*) images were taken of plants grown in an orchard at Los Lunas, NM, USA. Rice plants (*O. sativa*) were grown in field conditions or the greenhouse (*O. rufipogon*) and managed according to practices outlined in (*53*).

Generation of interspecific *pl* mapping population in Brinjal eggplant (*S. melongena*)

The prickleless Brinjal eggplant *S. melongena* 8104, hereafter MEL5, and the prickled eggplant wild relative *S. insanum* SLKINS-1, hereafter INS1 (*54*, *55*), were selected as founding parents to map *pl*. Advanced backcrosses (BC3) of MEL5 with introgressions from INS1 were obtained as part of a previous project (*45*).The parents, their interspecific F1 hybrid, and 90 BC3 advanced backcrosses were phenotyped for the presence of prickles at the cotyledon and 3^rd^-4^th^ true leaf development stages. Of these, 19 BC3 advanced backcrosses with prickles and 71 without prickles where genotyped with the high-throughput eggplant SPET platform (*56*, *57*) to associate the presence of prickles with shared introgression intervals. After identifying the most promising genomic region, the prickled advanced backcross material with the shortest shared introgressed interval (BC3-33-3-1) was selfed to obtain a segregating BC3S1 population, which was used for fine mapping.

Mapping of *pl* in Brinjal eggplant

A total of 622 BC3S1 seeds were germinated in growth chambers and individuals were screened for prickles two weeks post-germination. After one month, seedlings were transplanted to a greenhouse where they were phenotyped again. DNA from leaves was extracted following the SILEX protocol (*58*) and DNA yield and quality were measured spectrophotometrically using a NanoDrop™ ND-1000 (Thermo Scientific, Waltham, MA, USA). DNA integrity was checked by electrophoresis on an agarose gel (0.8%) (Condalab, Madrid, Spain) and diluted with ultra-pure water to a final concentration of 50 ng/µL.

Subsequently, primers were designed for the INS1 introgression based on the genome position of target SNPs used to perform SPET in eggplant (*56*, *57*) and the v3.0 eggplant genome sequence (*59*). To further delimit the ~2.5 Mb *pl* interval and identify recombination breakpoints, we performed PCR genotyping by HRM (High Resolution Melting) genotyping (*60*). Primers used for HRM genotyping are listed in **Table S9**. PCR reactions were performed on a Roche LightCycler® 480 System (Roche Diagnostics, Rotkreuz, Switzerland) using the MasterMix qPCR No-ROX PyroTaq EvaGreen 5x for HRM (CMB-Bioline, Madrid, Spain). Two microliters of the diluted DNA sample were mixed with 0.3 µL (10 µM) of each primer, 2 µL of MaterMix qPCR qPCR No-ROX PyroTaq EvaGreen 5x for HRM and ultra-pure water until a final volume of 10 µL. The PCR reaction conditions were as follows: an initial a pre-incubation step at 95°C for 15 min followed by 55 cycles of denaturation at 95°C for 10 s, annealing at 60°C for 15 s and elongation at 68°C for 15 s. A melting curve analysis (60°C to 95°C) was performed after amplification to check specificity of the reaction. The HRM generated data were analysed using the LightCycler 480 Software release 1.5.1.62 via T_m_ values and melting curve shapes. MEL5 and INS1 parents were used as a control to detect differences between homozygote and heterozygote genotypes. The *pl* interval was thereby narrowed to a region between SNP13 and SNP14 markers, corresponding to the 105,450,271-105,543,990 bp position of chromosome 6, with a total size of 93,719 bp.

RT-qPCR of PL in Brinjal eggplant (*S. melongena*)

Total RNA was isolated from the leaves of prickled and prickleless Brinjal eggplant (*S. melongena*) and prickled *S. insanum* using 700 µL of Extrazol® EM300 (Blirt DNA, Gdansk, Poland), according to the manufacturer’s specifications. Integrity was checked by 1% agarose gel electrophoresis, and purity and quantity were determined spectrophometrically using a NanoDrop™ ND-1000 (Thermo Scientific, MA, USA). Total RNA (1 µg) was treated with DNase I (RNase-free) (Thermo Scientific, MA, USA) and used as template in a FastGene Scriptase Basic cDNA Synthesis kit reaction (Nippon Genetics Europe, Düren, Germany) primed with Oligo dT. RT-qPCR was performed using a Roche LightCycler® 480 System (Roche Diagnostics, Rotkreuz, Switzerland) thermocycler using qPCR MasterMix No-ROX PyroTaq EvaGreen 5x for HRM (CMB-Bioline, Madrid, Spain). The PCR program consisted of an initial step of pre-incubation denaturation at 95°C for 15 min followed by 45 cycles of denaturation at 95°C for 15 s, annealing at 60°C for 20 s and extension at 72°C for 20 s. Melt curve analysis was performed to evaluate and confirm the specificity of the PCR reactions. An endogenous control, the eggplant housekeeping CAC gene (Clathrin adaptor complex medium subunit, SMEL_008g297560.1.01) was used as a normalization reference (*61*). Gene transcript expression level was analysed according to the relative quantitative accumulation by the 2^(-ΔΔCt)^ method (See **Table S9** for primers). A two-tailed Student’s t-test was used to determine statistical significance of inter-species differential expression.

RT-PCR of *PL* transcripts for splice isoform analysis in Brinjal eggplant and Gboma eggplant

Approximately 0.5 cm long leaf primordia were harvested from MEL5, INS1, and a line harboring a minimal *PL* introgression from INS1 in the MEL5 background. Similarly staged tissue was also harvested from prickleless Gboma eggplant (*S. macrocarpon* PI 441914), prickled *SmacPL^HET^* individuals identified in a stock of Gboma eggplant (*S. macrocarpon* SOLA112), and prickled homozygous wild-type *SmacPL* individuals in an F2 population derived from a parental cross between *S. macrocarpon* PI 441914 and *S. macrocarpon* SOLA112. All tissues were collected from greenhouse grown plants at approximately 10:00-11:00 AM and flash frozen in liquid nitrogen in 1.5 mL microfuge tubes containing a 5/32 inch (~3.97 mm) 440 stainless steel ball bearing (BC Precision, TN, USA). Tubes containing tissue were placed in a -80 °C stainless steel tube rack and ground using an SPEX^TM^ SamplePrep 2010 Geno/Grinder^TM^ (Cole-Parmer, NJ, USA) for 2 min at 1440 RPM. Total RNA was extracted using TRIzol (Invitrogen, MA, USA) according to the manufacturer’s instructions for ground tissue. RNA was treated with DNase I and cleaned using an RNA Clean and Concentrator Kit (Zymo Research, CA, USA) according to the manufacturer’s instructions. Purity and concentration of the resulting total RNA was assessed using a NanoDrop One^C^ spectrophotometer (Fisher Scientific, MA, USA). Next, 1 µg of total RNA was used as input for Poly-T primed reverse transcription using the SuperScript IV VILO Master Mix (ThermoFisher, MA, USA) kit according to the manufacturer’s instructions. PCR amplification was done with 1 µL of cDNA and a primer concentration of 10 µM in a 20 µL reaction using the KOD One^TM^ PCR Master Mix (Toyobo, Osaka, Japan). Amplification was performed using a Mastercycler X50 thermocycler (Eppendorf, Hamburg, Germany) with an initial 2 min 98 °C denaturation step, 35 cycles consisting of 10s denaturation at 98 °C, 10 s annealing at 55 °C, and 10 s extension at 68 °C, followed by a final extension step of 2 min at 68 °C. Equal volumes (5 µL) of the resulting PCR product were loaded onto a 2% biotechnology grade Agarose I (VWR International, PA, USA) gel prepared with 1X TBE buffer and 0.0025% ethidium bromide. Electrophoresis was performed in an Owl^TM^ D3-14 electrophoresis box (Thermo Scientific, MA, USA) containing 1X TBE buffer for 45 min at 130 V delivered from an Owl^TM^ EC 300 XL power supply (Thermo Scientific, MA, USA). The electrophoresis results were visualized under UV light using a Bio-Rad ChemiDoc^TM^ XRS+ (Bio-Rad, CA, USA) imaging platform and ImageLab^TM^ (Bio-Rad, CA, USA) software. Relevant primer sequences are listed in **Table S9.**

To determine the identity and relative abundance of transcript isoforms across the different genotypes, PCR products were cloned using a StrataClone Blunt PCR Cloning Kit (Agilent, CA, USA) according to the manufacturer’s instructions. PCR was conducted on single colonies swabbed into 10 µL sterile water and a 1 µL aliquot of the suspended culture was used as a PCR reaction template using 10 µM M13F/R primers with KOD One^TM^ PCR Master Mix. PCR amplification was performed with a 2 min 98 °C denaturation step, 35 cycles consisting of 10 s denaturation at 98 °C, 10 s annealing at 55 °C, and 10 s extension at 68 °C and followed by a final extension step of 2 min at 68 °C using a Mastercycler X50 thermocycler. PCR products were purified using 1.8X volume Ampure XP beads according to the manufacturer’s instructions and submitted for Sanger sequencing (Azenta Genewiz, NJ, USA).

Co-segregation and mapping of *pl* in Scarlet eggplant (*S. aethiopicum*) and Gboma eggplant (*S. macrocarpon*)

The *SaetPL* allele co-segregation analysis in Scarlet eggplant was performed on an F2 population derived from an intraspecific cross between the prickled accession SC102 and the prickleless accession PI 424860. Field grown plants were phenotyped for the presence/absence of prickles and young leaf tissue was collected and DNA extracted using the cetrimonium bromide (CTAB) method (*62*). Primers (see **Table S9**) were designed to amplify the exonic region of *PL* harboring the mutant *Saetpl* deletion allele identified in the genome of the prickleless parental accession. PCR reactions were prepared using 1 µL of DNA and 10 µM primers in a 10 µL reaction volume with KOD One^TM^ PCR Master Mix (Toyobo, Osaka, Japan). Amplification was performed on a Mastercycler X50 thermocycler (Eppendorf, Hamburg, Germany) with a 2 min 98 °C denaturation step, 35 cycles consisting of 10 s denaturation at 98 °C, 10 s annealing at 55 °C, and 10 s extension at 68 °C, followed by a final extension step of 2 min at 68 °C. PCR products were purified using a 1.8X volume of AMPure XP beads (Beckman Coulter, CA, USA) and submitted for Sanger sequenced (Azenta Genewiz, NJ, USA) for *SaetPL* genotyping. Genotypes were then associated with prickle phenotypes. Chi-squared goodness of fit tests were performed with GraphPad.

For *SmacPL* in Gboma eggplant the same initial co-segregation analysis was performed as was done for Scarlet eggplant, above (see **Table S9** for primers). Multiple F2 populations derived from an intraspecific cross between prickleless Gboma eggplant (*S. macrocarpon* PI 441914) and prickled Gboma eggplant (*S. macrocarpon* SOLA112) were generated. QTL-Seq was performed on a single F2 population made up of the progeny of a single prickled F1 parent. From a population of 132 individuals, the DNA from a random selection of 25 prickleless and 31 prickled F2 individuals, along with 8 prickleless parental accession individuals (*S. macrocarpon* PI 441914) was extracted from young leaf tissue using a DNeasy Plant Pro Kit (Qiagen, Hilden, Germany) according to the manufacturer’s instructions for high-polysaccharide content plant tissue. Tissue used for extraction was ground using an SPEX^TM^ SamplePrep 2010 Geno/Grinder^TM^ (Cole-Parmer, NJ, USA) for 2 min at 1440 RPM. Sample DNA (1 µL assay volume) concentrations were quantified with Qubit 1X dsDNA HS buffer (ThermoFisher, MA, USA) on a Qubit 4 fluorometer (ThermoFisher, MA, USA) according to the manufacturer’s instructions. Separate pools were made for the parental sample, the prickled F2 individuals, and the prickleless F2 individuals, with an equivalent mass (3 µg) of DNA pooled from each individual. Next, the DNA pools were purified using a 1.8X volume of AMPure XP beads (Beckman Coulter, CA, USA) and the DNA concentration and purity assayed by Qubit and a NanoDrop One^C^ spectrophotometer (Fisher Scientific, MA, USA), respectively.

Paired-end sequencing libraries for QTL-Seq analysis were prepared using >1 µg of pooled DNA with a KAPA HyperPrep PCR-free kit (Roche, Basel, Switzerland) according to the manufacturer’s instructions. Indexed libraries were pooled for sequencing on a NextSeq 2000 P3 chip (Illumina, CA, USA). Mapping was performed using using the end-to-end pipeline implemented in the QTL-Seq software package (v2.2.4, <https://github.com/YuSugihara/QTL-seq>) (*63*) with reads aligned against the *S. macrocapron* (Smac3, PI 441914) genome assembly. After QTL-Seq analysis revealed a second interval, *pl2*, an additional co-segregation analysis was performed across multiple F2 populations, genotyping *SmacPL* and the *pl2* locus on chromosome 4 identified by QTL-Seq. For the *pl2* interval, an A-to-T SNP at position Chr4:20,401,670, which modifies a DraI restriction site, was used to as the basis for a cleaved amplified polymorphic sequences (CAPS) genotyping assay. Following PCR amplification using the same conditions described for the *SaetPL* genotyping above (see **Table S9** for primer details), 5 µL of PCR product was added to a 10 µL reaction containing 0.2 µL DraI (New England BioLabs, MA, USA) and 1 µL rCutSmart^TM^ Buffer (New England BioLabs, MA, USA) and incubated for 2-4 hours at 37 °C. The reactions were then loaded onto a 1% agarose gel and electrophoresed in an Owl^TM^ D3-14 electrophoresis box (Thermo Scientific, MA, USA) containing 1X TBE buffer for 30 min at 180 V delivered from an Owl^TM^ EC 300 XL power supply (Thermo Scientific, MA, USA). The electrophoresis results were visualized under UV light using a Bio-Rad ChemiDoc^TM^ XRS+ (Bio-Rad, CA, USA) imaging platform and ImageLab^TM^ (Bio-Rad, CA, USA) software. Genotypes assigned based on visualized PCR product cleavage patterns and associated with prickle phenotypes. Chi-squared goodness of fit tests were performed with GraphPad.

Genome assembly and scaffolding

Reference quality genome assemblies for *S. aethiopicum*, *S. macrocarpon*, *S. prinophyllum*, and *S. cleistogamum* (see **Table S6** for accession information) were generated using a combination of long-read sequencing (Pacific Biosciences, CA, USA) for contigging and optical mapping (Bionano Genomics, CA, USA) for scaffolding. High-molecular weight DNA used for sequencing was extracted from greenhouse grown 4 week-old seedlings germinated in 96-cell flats and dark treated for 48 hrs prior to flash freezing. High molecular weight DNA was extracted according to the method described in (*64*). Two PacBio Sequel IIe flow cells (Pacific Biosciences, CA, USA) were used for the sequencing of each sample (average read N50 = 11,221 bp, average coverage = 53X, average read QV = 83.28). Prior to assembly, we counted k-mers from raw reads with KMC3 (version 3.2.1) and estimated genome size, sequencing coverage, and heterozygosity with GenomeScope2.0 (*65*). Sequencing reads from each sample were assembled with hifiasm (*46*) exact parameters and software version varied between samples based on the level of estimated heterozygosity and are reported in **Table S4**. Post assembly, the draft contigs were screened for possible microbial contamination as described in (*64*).

Optical mapping (Bionano Genomics, CA, USA) was performed for each sample to facilitate scaffolding. Scaffolding with optical maps was performed using the Bionano solve Hybrid Scaffold pipeline with default parameters (<https://bionanogenomics.com/support/software-downloads/>). Hybrid scaffold N50s ranged from 75,028,200 bp to 107,486,353 bp (see **Table S4** for more detail including Bionano molecules per sample). High-throughput chromosome conformation capture (Hi-C) from Arima Genomics, CA, USA was performed for one sample, *S. prinophyllum,* to finalize scaffolding. 311,616,288 reads were integrated with the Juicer (v0.7.17-r1198-dirty) pipeline. Next, misjoins and chromosomal boundaries were manually curated in the Juicebox (v1.11.08) application. Chromosomes were named based on sequence homology, determined with RagTag (*66*) scaffold (v2.1.0, default parameters), with the phylogenetically-closest finished genome (see **Table S4** for details). Finally, small contigs (< 50,000 bp) with > 95% of the sequence mapping to a named chromosome were removed. Additionally, small contigs (< 100,000 bp) with > 80% of the sequence mapping to a named chromosome that contained one or more duplicated BUSCO genes, but no single BUSCO genes, were also removed using a python script. Using merqury (*47*) with the HiFi data, the final consensus quality of the assemblies was estimated as QV=51.1333 on average and a completeness of 99.2741% on average.

Genome annotation

The gene annotation pipeline involved several crucial steps. Initially, the quality of raw RNA-Seq reads underwent assessment using FastQC v0.11.9. Subsequently, reference-based transcripts were generated using STAR v2.7.5c (*67*) and Stringtie2 v2.1.2 (*68*) workflows. To refine the data, invalid splice junctions from the STAR aligner were filtered out utilizing Portcullis v1.2.0 (*69*). Orthologs with coverage above 50% and 75% identity were lifted from Heinz v4.0 (*48*) and Eggplant v4.1 (*17*) via Liftoff v1.6.3 (*49*) using parameters --copies,--exclude_partial and employing both Gmap version 2020-10-14 (*70*) and Minimap2 v2.17-r941 (*71*) aligners. In addition, protein evidence from several published Solanaceae genomes (*17*, *48*, *72*), and the UniProt/SwissProt database were utilized to support gene annotation. Structural gene annotations were generated through the Mikado v2.0rc2 (*73*) framework, leveraging evidence from the Daijin pipeline (*74*). Additionally, microsynteny and orthology to Heinz v4.0 and Eggplant v4.0 were assessed using Microsynteny and Orthofinder v2.5.2 (*75*). Correction of gene models with inframe stop codons utilized Miniprot2 (*76*) protein alignments from Heinz v4.0 and Eggplant v4.1. Furthermore, gene models lacking start or stop codons were adjusted by placing them within 300 base pairs of the nearest codon location using a custom python script.

For functional annotation, ENTAP v0.10.8 (*77*) integrated data from diverse databases such as PLAZA dicots (5.0) (*78*), Uniprot/Swissprot (*79*), TREMBL, RefSeq, Solanaceae proteins, and InterProScan5 (*80*) with Pfam, TIGRFAM, Gene Ontology, and TRAPID (*81*) annotations. Finally, the annotated data underwent a series of filtering steps, excluding proteins shorter than 20 amino acids, those exceeding three times the length of functional orthologs, proteins lacking assigned orthologs that have unknown function, and transposable element (TE) genes, which were removed using the TEsorter (*82*) pipeline.

The completeness of the gene models was determined by assessing single-copy orthologs through BUSCO5 (*50*) in protein mode, comparing against the solanales_odb10 database. Additionally, the presence or absence of a curated set of 180 candidate genes known to be crucial in QTL studies was examined. Genome annotation summary statistics are presented in **Table S5**.

DNA extraction, *PL* PCR amplification, and sequencing of *Solanum* species

For herbarium samples, tissue (~ 1-2 cm^2^) was excised with a scalpel from mounted herbarium specimens held in the Steere Herbarium Collection at the New York Botanical Garden, New York, USA (see **Table S6** for voucher information). Silica gel-preserved tissues were obtained from the Royal Botanic Garden Edinburgh. Tissue was obtained from species documented to be prickleless and samples were inspected for the absence of prickles. Likewise, tissue was also obtained from closely-related prickled sister species for sequence comparison. In total, 8 putative loss-of-function coding or splice-site mutations were identified in herbarium samples across 7 species. Two distinct mutations were identified in separate *S. schimperianum* specimens. For technical reasons, it was not possible to survey the complete exonic space of all genes - primer binding site divergence and low DNA sample quality precluded complete survery exonic survey in most cases. Among prickleless herbarium specimens in which a complete exonic survery was possible, 7 lacked an obvious putative loss-of-function coding or splice-site mutation. Among the remaining prickleless herbarium material with incomplete exonic sequence coverage, 22 specimens had no detectable putative loss-of-function coding or splice-site mutations. However, this does not preclude the possibility that *PL* mutations exist in these samples. Such mutations could lie in unsampled regions of the gene body or in non-coding regions. In addition, other loci (i.e. *pl2* in *S. macrocarpon*) may also contribute to unexplained prickle losses in *Solanum*. Meanwhile, across 30 sampled prickled sister taxa, none of the reported loss-of-function *pl* alleles were found and no other putative loss-of-function coding or splice-site mutations identified.

To extract DNA from herbarium samples, ~25 mg of tissue was added to a 2 mL microfuge tube containing a 5/32 inch (~3.97 mm) 440 stainless steel ball bearing (BC Precision, TN, USA) and ground for 2 min at 1440 RPM in an SPEX^TM^ SamplePrep 2010 Geno/Grinder^TM^ (Cole-Parmer, NJ, USA). Before beginning the extraction the bench area and pipettes were cleaned with a 10% bleach solution to reduce the risk of environmental DNA contamination. DNA extraction was performed using a modified version of the CTAB/STE-based extraction methods described in (*83*) and (*84*). First, 1 mL of freshly-prepared STE (0.25 M sucrose, 0.03 M Tris pH 8, 0.05 M ethylenediaminetetraacetic acid [EDTA]) was added to the ground tissue and vortexed for 5 s. The sample was then centrifuged at 2000 x g for 10 min and the wash and centrifugation in STE buffer was repeated once. Next, 1 mL 2X CTAB and 4 µL of 1 M dithiothreitol (DTT) were added to the precipitated tissue and incubated with gentle rotating agitation in a hybridization oven for 1 hr at 65 °C. Following incubation, organic extraction was performed by addition of 750 µL of 24:1 chloroform/isoamyl alcohol and the samples were inverted 10 times. Phase separation was achieved by centrifugation for 10 min at 16,200 x g and ~900 µL of the aqueous phase was transferred to a new tube. To precipitate the DNA, 3 µL of 15 mg/mL GlycoBlue^TM^ coprecipitant (ThermoFisher Scientific, MA, USA) was added and well-mixed followed by 600 µL of chilled -20 °C isopropanol. DNA was allowed to precipitate at -20 °C for 1 hr. After precipitation, the samples were centrifuged at 20,000 x g for 30 min in a centrifuge chilled to 4 °C. The resulting DNA pellet was washed once with 70% ethanol and re-suspended in 50-100 µL 0.1X TE pH 8 buffer.

Because DNA form archived dried plant tissues is often fragmented (*83*), an exon screening based approach was taken to identify mutations in the CDS or immediately flanking splice acceptor/donor sites. Primers were designed for intronic regions ~20-30 bp surrounding exons based on the sequence of *PL* from closely-related species for which whole-gene amplification of *PL* was possible due to the availability of intact DNA isolated from fresh, living tissue (see **Table S9** for primers). PCR amplification was performed with 1-2 µL of DNA, primers at 10 µM, in a 10 µL reaction using the KOD One^TM^ PCR Master Mix (Toyobo, Osaka, Japan). Amplification was performed using on a Mastercycler X50 thermocycler (Eppendorf, Hamburg, Germany) with a 2 min 98 °C denaturation step, 35-40 cycles consisting of 10 s denaturation at 98 °C, 10 s annealing at 48-62 °C, and 10 s extension at 68 °C, followed by a final extension step of 2 min at 68 °C. PCR products were purified using 1.8X volume Ampure XP beads (Beckman Coulter, MA, USA) according to the manufacturer’s instructions. For high-yielding PCR reactions, the PCR products were directly Sanger sequenced (Azenta Genewiz, NJ, USA). For low-yielding PCR reactions, the PCR products were blunt cloned and sequenced as done for the PL transcript splicing isoform analysis described above. Sequences were then aligned (Geneious alignment) to either Brinjal eggplant *PL* gene body and screened for mutations using Geneious version 2022-07-07 software (see **Table S7**).

For the genotyping of *PL* in prickleless species for which fresh tissue was available, a CTAB-based method was used to obtain DNA (*62*). Primers (see **Table S9**) anchored in the first and final exon of *PL* were used to PCR amplify the entire gene body. Touchdown PCR amplification was performed using on a Mastercycler X50 thermocycler (Eppendorf, Hamburg, Germany) with an initial 2 min 98 °C denaturation step, 16 cycles consisting of an initial 10 s annealing at 68 °C thereafter descending 1 °C/cycle, a 1 min extension at 68 °C, and a 10 s 98 °C denaturation. Next, the reaction proceeded through 14-19 cycles of annealing for 10 s at 60 °C, extension for 1 min at 68 °C and 10 s denaturation at 98 °C followed by a final extension of 2 min at 68°C. PCR products were purified using 1.8X volume Ampure XP beads (Beckman Coulter, MA, USA) according to the manufacturer’s instructions. PCR products were then blunt cloned, as described above, and whole-plasmid sequenced (Plasmidsaurus, OR, USA) to detect variants across the *PL* gene body. In all 4 such cases, candidate *PL* mutations were identified. See **Table S7** for identified exonic *PL* mutations in prickleless species and the sequences of aligned prickled sister taxa).

Analysis of published mapping and RNA-Seq data from rose

The major QTL for prickle formation on rose stems was previously mapped on chromosome 3 using a mapping population derived from a cross between of *R. chinensis* ‘Old Blush’ (OB) and *R. wichurana* Basye’s Thornless (BT) (*9*, *85*). All genes in the Prickles QTL interval were *de novo* annotated in the genome of *R. wichurana* BT using the gene prediction tool AUGUSTUS (*86*)*.* Predicted annotations were refined by mapping the OB protein to the BT genomic regions with Exonerate (*87*)*.* Synteny is based on the match found by Exonerate. Previously published RNA sequencing data were used to examine expression of the LOG homolog contained within the rose mapping interval. Reads were trimmed using trimmomatic v0.39 (*88*) and then mapped to the Rosa chinensis genome using STAR v2.7.5c (*67*). Read pileups at 1 bp resolution were visualized by converting resulting bam files to bigwig format.

Virus induced gene silencing (VIGS) in rose

The TRV constructs pYL192 (TRV1) and pYL156 (TRV RNA2) were described in (*25*). A 300 bp sequence between nucleotide 49 and 348 after start codon of the *LOG* gene was synthesized (idtDNA.com), then cloned into pYL156 using EcoRI and BamHI restriction sites (see **Table S9** for sequence of insert). The integrity of the resulting LOGpTRV2 construct was then confirmed by sequencing. pYL192 (TRV1) and LOGpTRV2 were transferred into *Agrobacterium tumefaciens* EHA105 and then used for rose transformation. Agrobacteria harboring pYL192 (TRV1) or LogpTRV2 were plated on YEB medium containing 50 mg/L Kanamycin and 25 mg/L Rifampicin, and then grown for three days at 28°C. Agrobacteria were then collected in infiltration medium (YEB supplemented with 10mM MgCl2, 10mM MES, 200µM acetosyringone) at a final concentration of OD_600_=1.5.

After 2-3 h incubation at room temperature, *Rosa x hybrida* 'Rouge Meilland' cuttings were transfected by vacuum infiltration as follows. Three-month-old cuttings were immersed in a solution containing agrobacteria harboring pYL192 (TRV1) and LOGpTRV2 (1:1 ratio) supplemented with 300µL/L Silwet. Vacuum was maintained for 1 min at -0.04mPA twice with agitation in between. Cuttings were then planted in soil and grown in a growth chamber at a temperature of 17/24 °C (night/day) with a photoperiod of 8h/16h (night/day). Developing shoots were analyzed for prickle development three weeks after infiltration.

Identification of LOG mutations in Chinese date (*Ziziphus jujuba*) and giant spider-flower (

*Tarenaya hassleriana*)

Published genomes of Chinese date (*Z. jujuba*) were screened for mutations in LOG homologs. Candidate exonic loss-of-function mutations were identified in the cultivated spine-suppressed *Z. jujuba* cultivar Dongzao (GenBank assembly: GCA_000826755.1) and *Z. jujuba* cultivar Junzao (GenBank assembly: GCA_001835785.2) in a *LOG* homolog corresponding to LOC107411352. Mutations were not detected in this gene in the spiny wild relative *Z. jujuba* var. spinosa (GenBank assembly: GCA_020796205.1). For giant spider-flower a mutation was identified in a *LOG* homolog (LOC104826905) in the reference genome (GenBank assembly: GCA_000463585.1).

Phylogenetic analysis of angiosperm *LOG* homolog

*LOG* genes were identified across plant genomes using Orthofinder (*75*). for the chosen eleven species, the protein sequences of ninety-nine proteins were aligned using MAFFT v7.402 (*89*). The best scoring maximum likelihood tree was inferred using default parameters, with 1000 bootstrap replicates in RAxML v8.2.12 (*90*) via CIPRES (*91*). The tree was visualized in R using the ggtree package (*92*). The AtLOG_TypeIIIb (AT1G50575.1) protein sequence was used as an outgroup. Branch support was provided by 1000 replicate bootstrapping. The LOG sub-clades were designated after the naming convention used in (*93*).

Forest nightshade (*S. prinophyllum*) expression atlas

Washed and dried roots, leaves, flowers, fruits (with seeds removed), and developing prickles (~0.25-0.50 cm in length) were collected from 2 month-old Forest nightshade (*S. prinophyllum*) plants. All tissues were collected in 3-4 replicates, each from different plants. Total RNA was extracted using Quick-RNA MicroPrep Kit (Zymo Research) and treated with DNAse I (Zymo Research) according to the manufacturer’s instructions. RNA concentration and quality were analyzed using Thermo Scientific™ NanoDrop™ One^C^ Spectrophotometer. Libraries for RNA-sequencing were prepared by KAPA mRNA HyperPrep Kit (Roche, Basel, Switzerland). Paired-end 100-base sequencing was conducted on the NextSeq 2000 P3 sequencing platform (Illumina, CA, USA). Reads were trimmed using trimmomatic v0.39 (*88*) and then mapped to the Spri1 genome using STAR v2.7.5c and expression computed in transcripts per million (TPM). Expression data from the Forest nightshades in vegetative meristem generated in a previous study was processed in the same fashion (*94*). Gene expression data from ATHENA (https://athena.proteomics.wzw.tum.de/master_arabidopsisshiny/) for the *Arabidopsis* *LOG1* ortholog in equivalent tissues was downloaded and data for LOG homologs in tomato was downloaded from https://www.solpangenomics.com. Expressed genes were clustered phylogenetically based on alignment of their CDS sequences using the Clustal Omega function in Geneious (version 2022-07-07). A maximum likelihood tree using the Tamura-Nei model (*95*) was used for tree building.

Generation of CRISPR-Cas9-induced mutants and phenotyping

CRISPR Guide RNAs to target *PL* across Solanum species were designed using Geneious. The Golden Gate cloning approach as described in (*29*) was used to create multiplexed gRNA constructs. Plant regeneration and *Agrobacterium* *tumefaciens*-mediated transformation of *S*. *prinophyllum* and tomato were performed according to (*51*). The same methods were also used for *S. aethiopicum* and *S. cleistogamum* with two modifications. For *S. cleistogamum*, plant regeneration, the medium was supplemented with 0.5 mg/L zeatin instead of 2 mg/L and for the selection medium, 75 mg/L kanamycin was used instead of 200 mg/L. For *S. aethiopicum*, the protocol was the same as for *S. cleistogamum*, except the fourth transfer of transformed plantlets is done onto media supplemented with 50 mg/L kanamycin. Seed germination time in culture can vary between species and batches of harvested seeds. Typically, *S*. *prinophyllum* germination took 8 – 10 days and *S*. *cleistogamum* behaved similarly to tomato, germinating in 6 - 8 days.

First generation transgenic (T0) plants (N ≥ 3) were screened for prickle suppression phenotypes relative to non-transgenic regenerated control plants. DNA was extracted from T0 individuals using the CTAB method (*62*) and primers (see **Table S9**) were used to amplify the entire *PL* gene body, which was cloned, and whole-plasmid sequenced as was done for the pan-Solanum screening for mutations in the *PL* gene body. For each T0 individual, 5-8 clones were sequenced to verify the association of the prickleless phenotype and null multiallelic or monoallelic T0 phenotype. For *S. prinophyllum* and *S. lycopersicum*, CRISPR alleles advanced to the T1 generation showed similar phenotypes to the T0.

Scanning Electron Microscopy

For prickle imaging in barley (*Hordeum vulgare*), freshly isolated awn samples were fixed with 2% glutaraldehyde in 50 mM phosphate buffer pH 7.0 for 16h at 8°C. After two 5 min washes with distilled water, samples were dehydrated in an ascending ethanol series of 30%, 50% 70%, 90%, 100%, and a second 100% ethanol wash with each step lasting 10 min after which they were critical point dried in a Quorum K850 critical point dryer (Quorum Technologies Ltd., https://www.quorumtech.com). Dehydrated samples were placed onto carbon adhesive discs, gold coated in an Edwards S150B sputter coater (Edwards High Vacuum Inc., Burgess Hill, UK) and examined in a Zeiss Gemini300 scanning electron microscope (Carl Zeiss Microscopy GmbH, Jena, Germany) at 5 kV acceleration voltage. For scanning electron microscopy of rose (*Rosa*), *Solanum*, and rice (*Oryza sativa* and *O. rufipogon*) prickles, fresh tissue was adhered to stubs using non-conducting adhesive tape and imaged using a NanoScope JCM-7000 Benchtop SEM (JEOL, Tokyo, Japan).

Brix assay to measure fruit sugar content

From 6 Desert raisin (*S. cleistogamum*) plants, 12 fully ripened and dried fruits were randomly selected to determine their soluble sugar content (Brix). Fruits were briefly rehydrated by soaking in DI water for 60 minutes. Following rehydration, the fruit were patted dry and placed into a 50 mL tube. Rehydrated fruit were then crushed using a spatula. The slurry was centrifuged at 15,000 rpm for 5 min. The supernatant was drawn from the samples and diluted 1:30 to obtain a working volume of 300 µL. The Brix value (%) was quantified from three technical replicates with an ATAGO Palette digital Brix refractometer (ATAGO, Tokyo, Japan). See **Table S8** for data.

Cross-species *LOG* family co-expression conservation analysis

To calculate the co-expression conservation between tomato genes and *Arabidopsis thaliana* genes, gene family orthology information was downloaded from OrthoDB V11(*96*). Using one-to-one focal gene pairs between the two species, the degree to which co-expression is conserved between all *LOG* genes in each species was calculated (*97*). Briefly, a robust gold standard coexpression network for each species was sourced (*27*), which consists of coexpression networks built from hundreds of samples across dozens of experiments. For each Arabidopsis *LOG* gene, the top 10 genes it was coexpressed with were identified and used to predict the coexpression partners of all *LOG* genes in tomato. The degree to which the top coexpressed genes in *Arabidopsis* predicted the top coexpressed genes for a given *LOG* homolog in tomato yielded the co-expression conservation score, which is defined as the area under the receiver operatoring characterstic (AUROC) curve. This was then repeated in the other direction, taking the top 10 coexpression partners of each tomato *LOG* gene and predicting the coexpression partners of all Arabidopsis *LOG* genes. The two resulting AUROC statistic values are averaged, resulting in the final co-expression conservation scores.

Fig. S1.

**Prickles are morphologically similar.** (**A-C**) Scanning electron micrographs (SEMs) of prickles in rose (*Rosa* spp.) (A), Malevolence (*Solanum atropurpureum*) (B), and at an early stage of development in Forest nightshade (*Solanum prinophyllum*) (C).

Fig. S2.

**Characterization of *PL* alleles in Brinjal (*S. melongena*) and Gboma eggplant (*S. macrocarpon*).** (**A**) A second *Smelpl* allele in the re-sequenced accession GPE003520 removed exon 6 as evidenced by a lack of whole-genome sequencing reads mapped to this region. Reads mapping to the forward strand are in red reads mapping to the reverse strand are in blue. (**B**, **C**) Gene body diagrams illustrate the canonical and *PL* isoforms for common eggplant (A) and Gboma eggplant (B). Below are detected *PL* isoforms from the leaf tissue of plants homozygous for the indicated alleles. Fractions represent the proportion of isoforms of each type detected. (**D**) RT-qPCR analysis of *PL* expression in the leaves of wild eggplant (*S. insanum*) and common eggplant. The expression of two transcription factors nearby the *pl* mapping interval are also shown. Error bars reflect the standard deviation about the mean, Student’s two-tailed t-test, * *p* < 0.05, *n* ≥ 3. (**E**) *Saetpl* co-segregates with the prickleless phenotype in a Scarlet eggplant F2 mapping population. (**F**) Alleles generated from CRISPR-Cas9 genome editing of *SaetPL* in *S. aethiopicum* (accession PI 666076) (**G**) F2 population confirms co-segregation of *Smacpl* and *pl2* with the prickleless phenotype. Numbers within bars (E and G) indicate count totals of individuals within each genotype class.

Fig. S3.

**Identification of *PL* mutations in the spiny *Solanum*.** The phylogeny from Fig. 1B is re-produced at left and species for which all or part of *PL* were sequenced are shown. Sequences of the indicated regions are numbered according to their position in Table S6.

Fig. S4.

**Small prickles are occasionally observed on *pl^CR^* plants.** Bright-field images of leaves of Forest nightshade (*S. prinophyllum*) WT and *Spripl^CR^* plants. Arrowhead indicates the presence of a small prickle along a *Spripl^CR^* leaf vein.

Fig. S5.

**Trichome density and morphology is qualitatively unaffected in genome edited *Sclepl* mutants.** Scanning electron micrographs of the *S. cleistogamum* petiole in the fully-expanded fourth leaf just below the lamina-petiole boundary (*N* = 4 plants for each indicated genotype).
