# Supplementary figures and images for: Convergent evolution of plant prickles by repeated gene co-option over deep time
Source: Science. Author manuscript; Available in PMC 2024 Aug 7. (PMC11305333; doi:10.1126/science.ado1663)

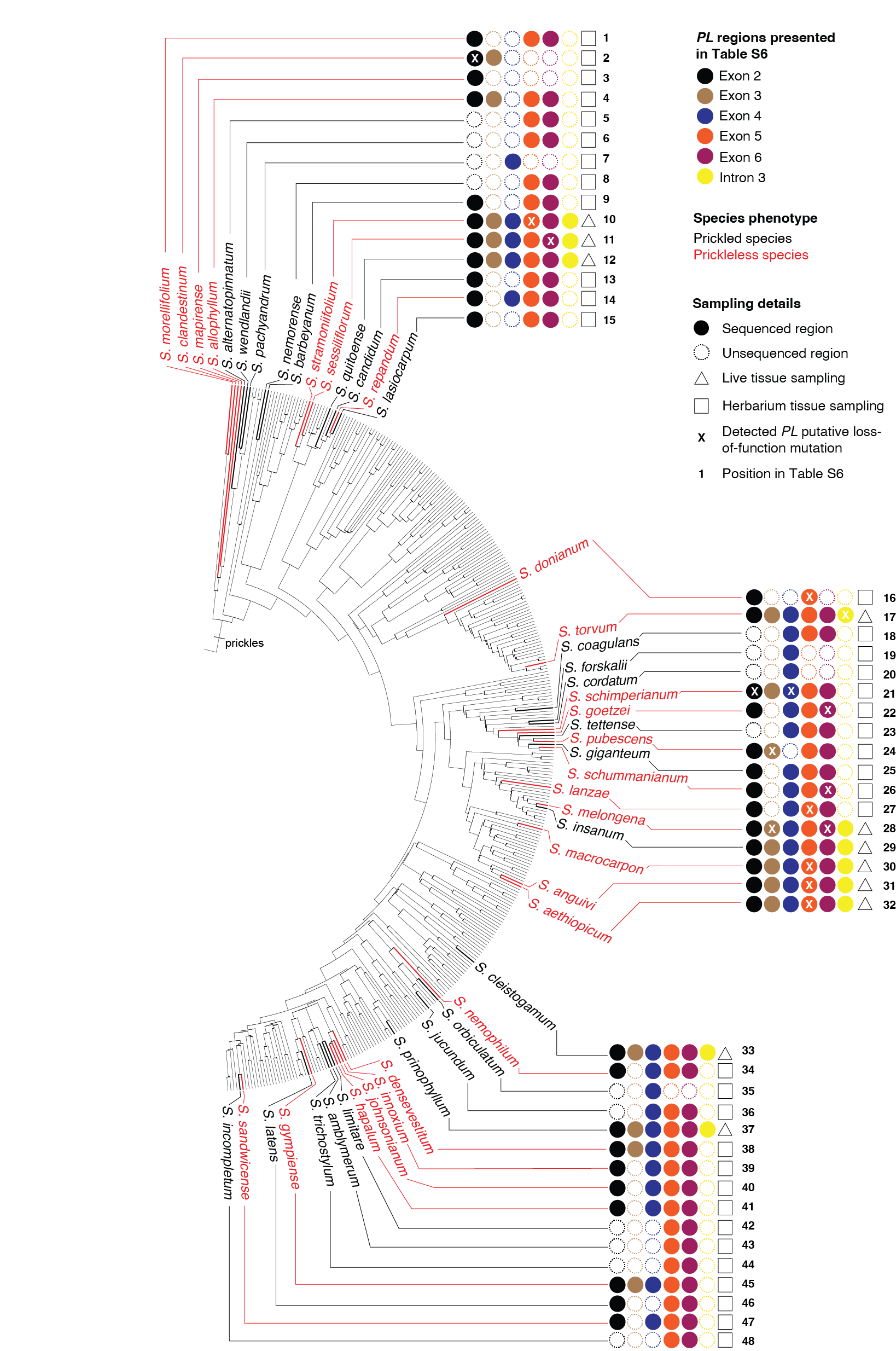

Supplement: Figure_S3 [file NIHMS2012043-supplement-Figure_S3.png]

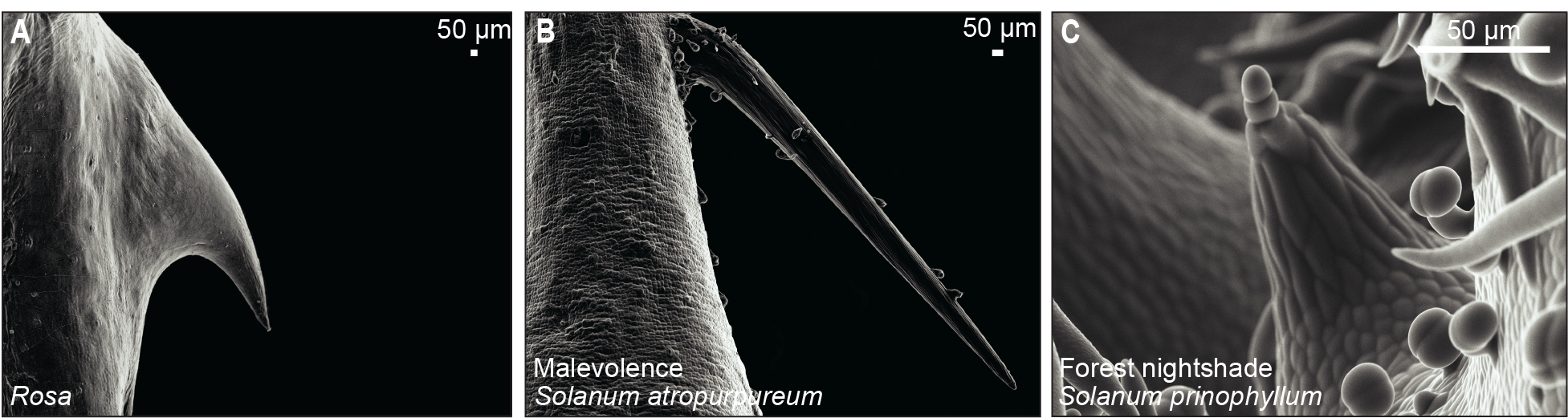

Supplement: Figure_S1 [file NIHMS2012043-supplement-Figure_S1.png]

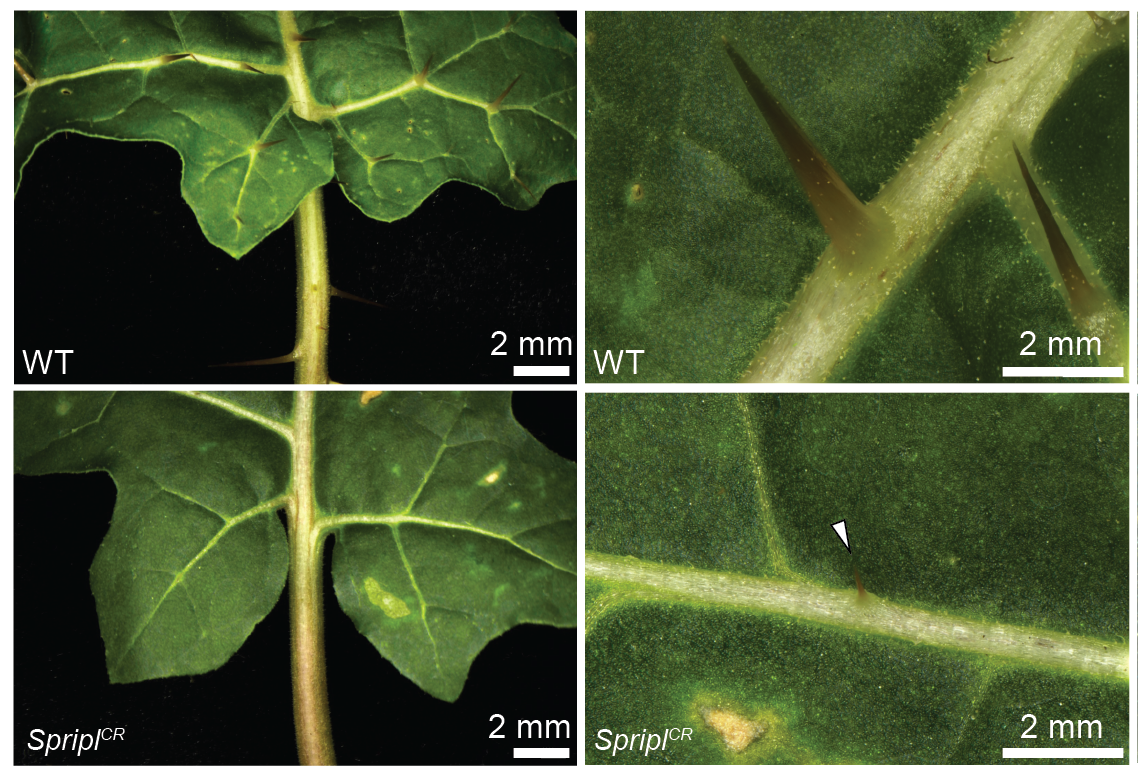

Supplement: Figure_S4 [file NIHMS2012043-supplement-Figure_S4.png]

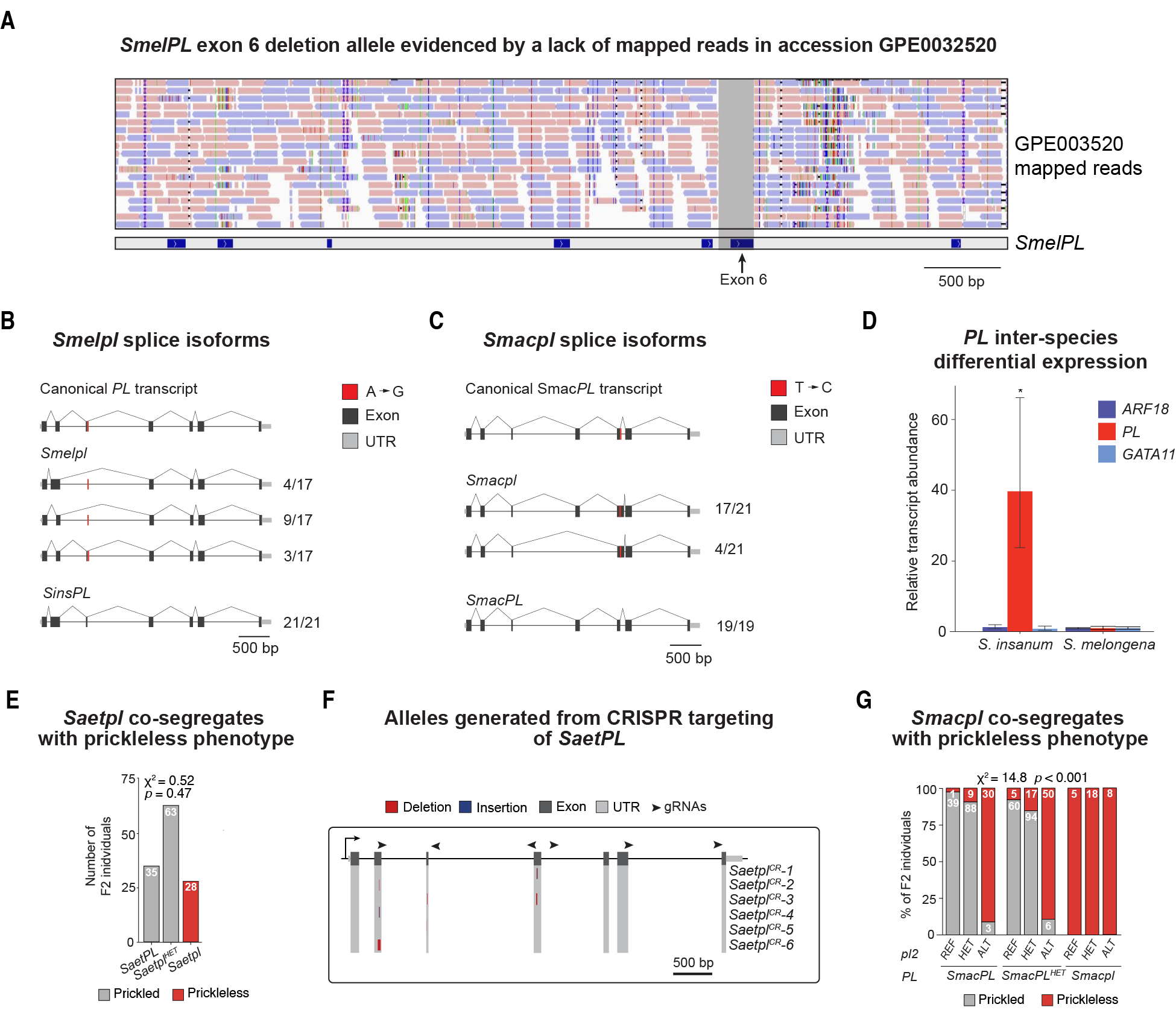

Supplement: Figure_S2 [file NIHMS2012043-supplement-Figure_S2.png]

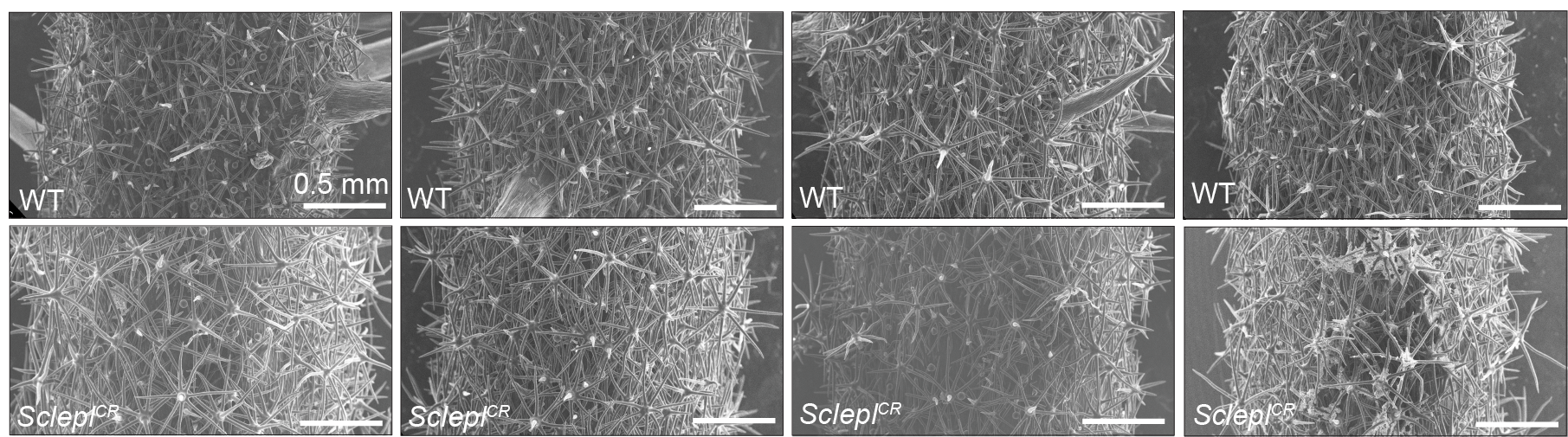

Supplement: Figure_S5 [file NIHMS2012043-supplement-Figure_S5.png]
